# Oxidative elemental cycling under the low O_2_ Eoarchean atmosphere

**DOI:** 10.1038/srep21058

**Published:** 2016-02-11

**Authors:** Robert Frei, Sean A. Crowe, Michael Bau, Ali Polat, David A. Fowle, Lasse N. Døssing

**Affiliations:** 1 Department of Geoscience and Natural Resource Management, University of Copenhagen, Copenhagen, Denmark; 2 Nordic Center for Earth Evolution, NordCEE, Denmark; 3 Departments of Microbiology and Immunology, and Earth, Ocean, and Atmospheric Sciences, University of British Columbia, Vancouver, Canada; 4 Department of Physics and Earth Science, Jacobs University Bremen, Bremen, Germany; 5 Department of Earth and Environmental Science, University of Windsor, Canada; 6 Department of Geology, University of Kansas, Lawrence, KS, USA

## Abstract

The Great Oxidation Event signals the first large-scale oxygenation of the atmosphere roughly 2.4 Gyr ago. Geochemical signals diagnostic of oxidative weathering, however, extend as far back as 3.3–2.9 Gyr ago. 3.8–3.7 Gyr old rocks from Isua, Greenland stand as a deep time outpost, recording information on Earth’s earliest surface chemistry and the low oxygen primordial biosphere. Here we find fractionated Cr isotopes, relative to the igneous silicate Earth reservoir, in metamorphosed banded iron formations (BIFs) from Isua that indicate oxidative Cr cycling 3.8–3.7 Gyr ago. Elevated U/Th ratios in these BIFs relative to the contemporary crust, also signal oxidative mobilization of U. We suggest that reactive oxygen species were present in the Eoarchean surface environment, under a very low oxygen atmosphere, inducing oxidative elemental cycling during the deposition of the Isua BIFs and possibly supporting early aerobic biology.

Sedimentary rocks from the 3.8–3.7 Ga Isua Greenstone Belt (IGB; Supplementary Fig. 1) in Greenland contain a vestige of the nascent Archean biosphere, recording the chemistry of the ocean-atmosphere system during the early proliferation of microbial life on Earth. Metasedimentary rocks from Isua contain carbon-13 depleted graphite—a diagnostic feature of biomass formed through autotrophic microbial activity, and one of the earliest indicators for life on Earth^1^,^2^. Likewise, deposition of oxide-facies Banded Iron Formations (BIFs), marine chemical sediments originally comprised of alternating layers of silica and Fe oxyhydroxides, may signal the advent of photosynthesis by 3.8–3.7 Ga. BIF are widely used as geochemical archives since they retain information on the composition and redox state of the seawater from which they formed. For example, the rare earth and yttrium element (REY) patterns of chemically pure (i.e., detritus-free) BIF, faithfully record seawater REY patterns. These patterns show that distinctive chemistries not recognized in any other material studied to date, such as overall LREE depletion relative to the HREE, positive La and Gd anomalies, and elevated Y/Ho ratios have characterized seawater since the early Archaean^3^,^4^,^5^. BIF deposition requires substantial concentrations of dissolved ferrous Fe (Fe^2+^) in seawater, implying anoxic deep oceans and a relatively low oxygen atmosphere^6^,^7^. Deposition of oxide-facies BIF results from the oxidation and precipitation of this Fe^2+^ as mixed valence Fe(oxy)hydroxides. Both oxygenic photosynthesis by cyanobacteria and anoxygenic photosynthesis with Fe(II) as the electron donor (photoferrotrophy) may have contributed to Fe(II) oxidation and BIF deposition. Though the precise oxidative mechanisms remain contested, emerging insight strongly implicates photoferrotrophy^8^,^9^,^10^ prior to the proliferation of cyanobacteria and the large-scale oxygenation of the ocean-atmosphere system around 2.4 Ga. Geochemical evidence now suggests the evolution of cyanobacteria as early as 3.3–2.9 Ga^11^,^12^,^13^, suggesting a possible role for oxygenic photosynthesis in BIF deposition after this time, but in the absence of reactive oxygen species (ROS) like free molecular oxygen (O_2_) or hydrogen peroxide (H_2_O_2_), the earliest BIFs would have been deposited exclusively through anoxygenic photosynthesis. Biological oxygenic photosynthesis is the largest source of ROS (O_2_) on the modern Earth, but low-level photochemical production of H_2_O_2_ might have played a more important role prior to the evolution of oxygenic photosynthesis. To test for a possible role of ROS during the deposition of Isua BIFs at 3.7–3.8 Ga, we analysed Cr isotopes and trace elements in a suite of rocks from Isua.

Recognizing the inherent difficulties associated with extracting primary information from rocks that have experienced high-grade metamorphism like at Isua, we turned to comparative analyses of depositionally and genetically related rocks with similar geologic histories. We then used proxies least likely to experience significant post-depositional disturbance. Chromium isotopes are fractionated through redox reactions and these redox reactions require at least traces of ROS^14^. The Cr isotopic composition of marine chemical sediments such as BIFs or carbonates can therefore record chromium redox cycling, and when fractionated, appear to indicate the presence and action of molecular oxygen or hydrogen peroxide. Importantly, high-temperature processes such as metamorphic reactions subsequent to deposition are unlikely to further fractionate Cr isotopes at the scale of whole rock hand samples which would require large-scale remobilization of poorly soluble Cr(III) species and thus the Cr redox proxy can be applied even to metamorphic rocks such as those from Isua.

## Results

**Figure 1:**
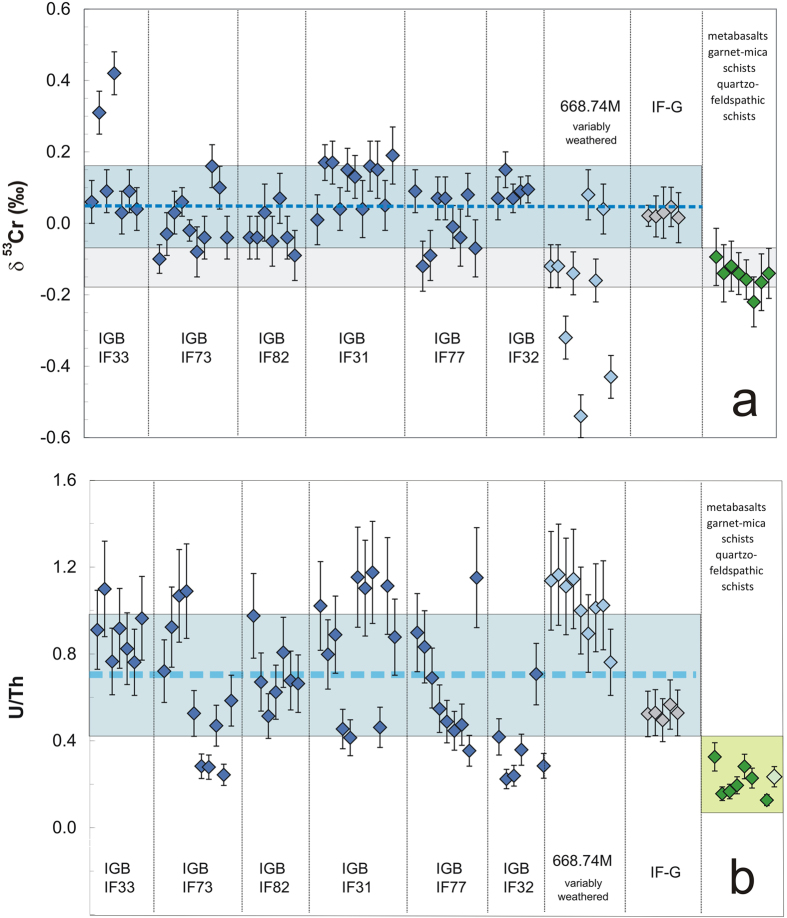
Cr isotope values and U/Th ratios for BIF samples from Isua. Cr isotope values (**a**) and U/Th ratios (**b**) of individual Fe and Si-rich mesobands of BIF samples and of associated metabasalts and metamorphosed clastic sediments from the Isua Greenstone Belt (data in Table S1). The grey-filled area in A delineates the δ^53^Cr as defined by magmatic rocks^15^. The dashed blue line and the blue-filled range in A denote the average δ^53^Cr value and the 1σ confidence interval, respectively, for all samples, excluding 97-Isua-1 668.74M. Error bars in A denote 2σ errors (Supplementary Table 1). Error bars in B reflect the 2σ reproducibility of the U/Th ratios. The dashed blue line and the blue-filled area in B denote the average U/Th value and the 1σ confidence interval (excluding 97-Isua-1 668.74N). Sample IF-G (grey-filled symbols) is an iron ore standard from the IGB^47^. The light green-filled symbol band in B mark the average, and the 1σ confidence interval of 59 least altered metabasalts and clastic metasediments from the IGB reported by^16^,^17^,^18^.

We find positive Cr isotope values (average δ^53^Cr = +0.05 +/−0.10 permil; δ^53^Cr = (^53^Cr/^52^Cr)_sample_/(^53^Cr/^52^Cr)_SRM 979_−1) × 1000, where SRM 979 denotes Standard Reference Material 979; Fig. 1a) in both the Fe and Si-rich mesobands of 7 compositionally distinct quartz-magnetite and magnesian iron formation samples (Supplementary Table 1; petrographical description and classification in supplementary information; Supplementary Figs 2,3) collected from the eastern portion of the Isua BIF (Western Greenland; supplementary information; Supplementary Fig. 1). This is a region of low strain characterized by minimal metamorphic recrystallization where the BIFs preserve primary depositional features and geochemical compositions (supplementary information). The preservation of primary chemistry is well demonstrated through the REY patterns, which carry the hallmark features, LREE depletion, superchondritic Y/Ho ratios, and positive La and Gd anomalies, of seawater precipitates (details in supplementary information; Supplementary Figs 4–6). The patterns also imply that these BIFs were largely free of detrital components suggesting that they can faithfully record seawater chemical signatures, despite subsequent metamorphism (supplementary information). Comparing the mean δ^53^Cr value of Isua BIFs to the mean of igneous rocks measured to date^15^ demonstrates fractionation of Cr isotopes at very high probability (*p* < 0.0001: Supplementary Table 2). Measurements of closely associated picritic and boninitic metabasalts^16^,^17^, as well as of clastic metasediments^18^ (Fig. 1a), from Isua, reveals that these rocks have δ^53^Cr compositions indistinguishable (*p* = 0.23; Supplementary Table 2) from the igneous silicate inventory, despite having a metamorphic history similar to that of the depositionally and genetically associated BIFs. Post-depositional hydrothermal fluids carrying positively fractionated Cr, likely sourced from the serpentinization of ultramafic rocks^19^ during tectono-metamorphic processes, could have percolated through the BIFs imparting the positively fractionated chromium signal we observe. However, the fact that a quartzo-feldspathic sample (462915; Supplementary Table 1) with a similarly low Cr concentration as the BIFs, preserves an igneous δ^53^Cr value of −0.12 +/−0.07% speaks against a large scale external source of the isotopically heavy chromium. We therefore argue that primary δ^53^Cr values have been preserved in the BIF throughout the metamorphic history of the Isua rocks. Positively fractionated Cr isotope signals from Isua are confined to the BIFs, indicating that this δ^53^Cr signal was inherited from ambient seawater during BIF deposition.

We also examined the distribution of U, which is rather immobile in its reduced tetravalent state but mobile when it is oxidized to U(VI). As U(IV) behaves similar to non-redox-sensitive Th(IV), epiclastic sedimentary rocks and the upper continental crust as such are characterized by coupled behaviour of U and Th, resulting in U/Th ratios well-below unity. In marked contrast, U and Th are decoupled in supergene oxic systems such as modern seawater, due to the predominance of U(VI) species. Hence, Phanerozoic marine chemical sediments that precipitated from oxic seawater show U/Th ratios that are considerably higher than those of clastic sediments and igneous rocks. Such a difference in U/Th ratios, therefore, signals differential behaviour of U and Th, which can be attributed to redox cycling of U as a likely result of oxidative weathering. In 6 of 7 BIF samples analysed, U/Th ratios are higher (*p* between <0.0001 to 0.35; Supplementary Table 2) than estimates for Archean crust^20^. As a whole, the Isua BIF including the IF-G BIF standard from Isua but excluding the weathered sample 97-Isua-1 668.74M carries a mean U/Th ratio of 0.70 ± 0.29. This ratio is much higher than that of Archean crust (U/Th = 0.26; *p* < 0.0001; Fig. 1b; Supplementary Table 2) and of the metabasalts and clastic metasediments associated with the Isua BIFs (Fig. 1b, Supplementary Table 1). Supporting evidence for U mobility during the formation of Isua sediments also comes from IGB schists for which high primary U/Th ratios were inferred from lead isotopes^21^.

**Figure 2:**
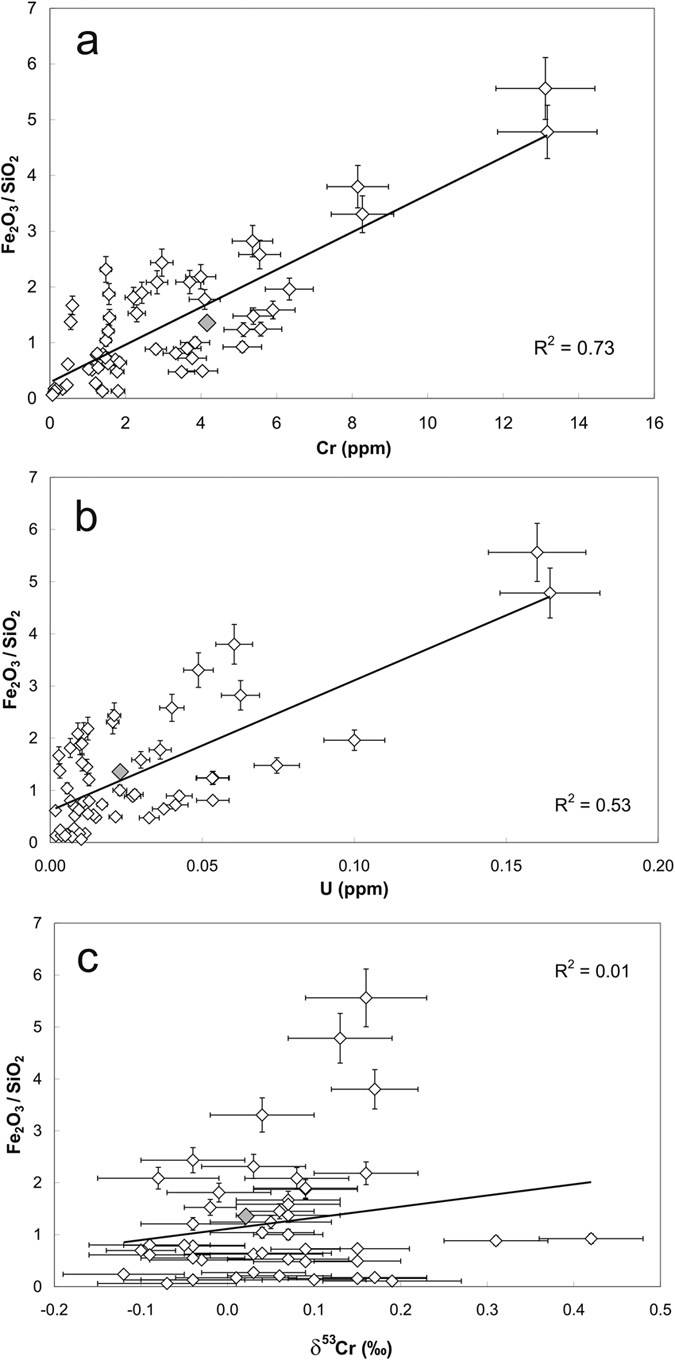
Selected major and trace element relationships in BIF samples from Isua. Relationships between Fe_2_O_3_/SiO_2_ ratios and Cr concentrations (**a**), U concentrations (**b**) and δ^53^Cr values (**c**) in mesobands from 7 tectonically least affected magnesian and quartz-magnetite BIF samples from the IGB. The grey-filled symbol marks the average of the IF-G standard. Error bars denote the conservative 10% 2σ reproducibility of the U and Cr concentrations, and the Fe_2_O_3_/SiO_2_ ratios, and the 2σ error of “n” repeated mass spectrometry runs of every sample in case of δ^53^Cr values (Supplementary Table 1). While the correlations between Fe_2_O_3_/SiO_2_, Cr and U are consistent with Cr and U sequestration involving Fe(oxyhydr)oxides through sorption and reduction processes, respectively, the heterogeneous Cr isotope signatures likely signify dynamics in the δ^53^Cr values of seawater.

Both Cr and U concentrations, but not δ^53^Cr values, in the different BIF mesobands are positively correlated with Fe_2_O_3_/SiO_2_ ratios (Fig. 2). This illustrates the close association of Cr and U to Fe minerals, reflecting their common scavenging by precipitating Fe (oxy)hydroxide precursors^22^. This is also illustrated by the pronounced correlation between U and Cr concentrations depicted in Supplementary Fig. 7. Together, these relationships argue against a redistribution of U and Cr during metamorphic overprinting. Instead, this correlation attests to the preservation of distinct δ^53^Cr and U/Th signals in the individual mesobands of each individual sample, and the same correlation is also defined by BIF hand sample specimens from different locations within the central tectonic domain of the IGB. This latter observation is consistent with between band Fe isotope heterogeneity in the Isua BIFs^23^, which indicates that rehomogenisation of Fe, and by inference other metals, by the amphibolite facies metamorphism that has affected the Isua rocks, was only effective at small scales, leaving signals intact between bands and hand-samples. An exception to this might be illustrated by sample 97-Isua-1 668.74M which is the only sample that experienced pervasive secondary hematitisation due to exposure to intense modern glacier meltwater percolation. Some mesobands of this drill core sample do show δ^53^Cr values which are more negative than the igneous silicate inventory value (Fig. 1; Supplementary Table 1) which we interpret to result from modern, meltwater-induced oxidative mobilization of isotopically heavy Cr from the BIF, leaving isotopically light Cr signatures behind.

## Discussion

A robust test for the presence of ROS in the 3.7 Ga ocean atmosphere system rests on the fidelity with which our Cr and U data record primary seawater signatures as opposed to tracking subsequent modifications through hydrothermal processes during BIF deposition, post-formational BIF alteration, metamorphic overprinting, or recent oxidative weathering. Hydrothermal alteration (i.e. serpentinization, hydration), indeed, shifts the Cr isotope compositions of oxidatively weathered ultramafic rocks to heavier values^19^. Such isotopically heavy δ^53^Cr values in altered ultramafic rocks are associated with various secondary and metamorphic Cr-rich minerals and thereby support previous results by Schoenberg and co-workers^15^. These isotopically heavy secondary Cr minerals likely incorporate Cr(III) as the product of Cr(VI) reduction in mineral-forming fluids. Such a process should be accompanied then by an associated shift (Rayleigh distillation) of the residual Cr in the fluid towards heavier δ^53^Cr values^24^. Redistribution of Cr resulting in isotopically heavy Cr signatures in granitic^25^, basaltic^26^,^27^ and ultramafic^28^ modern and ancient rocks, point to dynamic and complex redox cycling of Cr in some of the Earth’s crustal and near-surface environments. A key point here, however, is the requirement of redox processes, and therefore ROS, in both imparting Cr isotope fractionation and controlling the magnitude of Cr redistribution and the expression of isotopic fractionation at the scale of bulk rock samples. We can evaluate the extent of such alteration for the Isua BIFs we analyzed by combining diverse geochemical and petrological data available from Isua rocks with our new data.

Zinc-isotopes in a variety of rocks from Isua reveal a pronounced depletion in isotopically heavy Zn with respect to the igneous average pointing to a scenario whereby Isua rocks were permeated by carbonate-rich, high-pH reducing hydrothermal solutions at temperatures between 100–300 °C^29^. Unlike in the studies mentioned above, the BIFs and associated volcanic and sedimentary rocks in the least altered and least deformed central tectonic domain of the eastern ISB do not exhibit features of secondary hydrothermal overprinting (see Supplementary information for details). Our detailed petrographic inspection did not reveal secondary carbonates, which are the likely hosts for Zn species (and potentially also Cr(VI) species) under the low-temperature, highly alkaline hydrothermal conditions indicated previously^29^. Likewise, the absence of Ce-anomalies and the preservation of LREE-depleted, seawater-type REY patterns with strong positive Eu anomalies, which we report for the BIFs studied herein (Supplementary information) argue against large-scale permeation of BIF by post-formational hydrothermal solutions in this part of the ISB. Such hydrothermal fluids, if they had been present, would have likely caused an enrichment of the LREE signatures of the BIF samples, a feature we do not observe in our samples (see Supplementary information). Our analyses did not identify (despite intensive optical and electron microscopic investigations) secondary U-bearing phases (such as apatite, monazite etc.) which would be expected following U redistribution by hydrothermal fluids. This is corroborated by Pb isotope results of BIFs from within the same tectonic domain, which indicate a closed U-Pb system since BIF deposition^30^. Likewise, Sm–Nd isotopic relationships between individual layers reveal two distinct REE sources; seafloor-vented hydrothermal fluids (εNd (3.7 Ga)∼+3.1), and ambient surface seawater. The latter attained its composition by erosion of parts of the protocrustal landmass (εNd(3.7 Ga) ∼ +1.6)^30^. A third (i.e. post-depositional) REE source is not apparent. Finally, other sedimentary and volcanic rocks within the same tectonic domain as the BIFs studied herein preserve igneous inventory δ^53^Cr values and typical crustal U/Th ratios, a feature that speaks strongly against pervasive secondary alteration or metamorphic redistribution of these elements.

In an approach similar to that used by Li and co-workers^31^ for the 3.46 Ga Marble Bar chert (MBC) from the Pilbara Craton, NW Australia, we estimated the concentration of U in the Eoarchean Isua seawater basin, based on the U concentrations measured in BIF samples from Isua. Uranium concentrations in the Isua BIFs are, on average, around four times higher (~27 ppm) than in the MBC (~6 ppm; excluding three samples with anomalously high concentrations^31^), but measured U/Th values agree well (Isua: 0.70 ± 0.29; MBC: 0.67 ± 0.17^31^). The higher U concentrations in the Isua BIF samples may reflect the affinity of U(VI) (and Th(IV)) for sorption onto Fe(III) oxyhydroxides, i.e. they reflect the higher Fe_2_O_3_ concentrations in the Isua BIFs (5.6–77.6 wt%) relative to the cherts of MBC (0.06–9.7 wt%)^31^ shown in Fig. 2b. The Isua BIFs (and likewise the MBC) are not 100% pure detritus-free chemical sediments and minor detrital contamination, for example, is indicated by Al_2_O_3_ concentrations between 0.02 to 1.02 wt% (Supplementary Table 1) for the individual BIF mesobands. This suggests that in both sample sets, the pure marine chemical sediment-endmember showed an even higher U/Th ratio than those measured in the Isua BIF and Marble Bar chert samples. The U/Th ratios above the average crustal value of ~0.26 in the chemical sediments imply a relative enrichment of dissolved U relative to Th in the seawater from which the chemical sediments precipitated. The required decoupling of U and Th can only result from oxidative mobilization of U in the Earth’s surface system.

**Figure 3:**
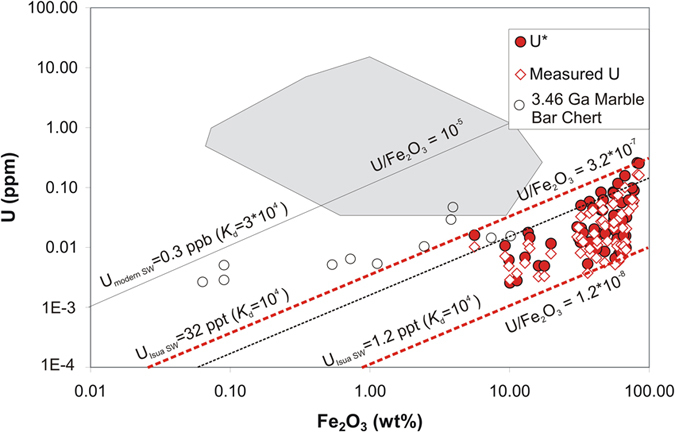
U versus Fe_2_O_3_ diagram with BIF samples from Isua. Data from Isua are compared with U and Fe_2_O_3_ contents of the 3.46 Ga Marble Bar Chert (MBC)^31^ samples, and with Phanerozoic cherts from three different tectonic settings (grey filled area; data sources in Li *et al.*^31^. U* depict authigenic U concentrations in the BIFs (measured U corrected for 3.7 Ga decay and for detrital share using Th concentrations and an average crustal U/Th of 0.25). Using a conservative U *K*_d_ value of 10^4^ between Fe(III) hydroxides and aqueous solution^32^,^33^, very low seawater U (U_SW_) concentrations of between 1.2 to 32 ppt (dashed red lines) are estimated for the 3.7 Ga seawater. The dotted black line is the reference MBC 3.42 Ga seawater U = 20 ppt line^31^. In conjunction with the Cr isotope data presented herein (Fig. 1a) and the positive U-Cr correlation (Supplementary Fig. 7) defined by the Isua BIF samples, we hypothesize that very low atmospheric oxgen levels were sufficient to mobilize small amounts of redox sensitive U and Cr through oxidative weathering conditions prevailing on land at 3.7 Ga.

Based on authigenic (detrital and decay-corrected U (U*; Supplementary Table 1; details in Li *et al.*^31^) and Fe_2_O_3_ concentration relationships in the MBC samples, and a conservative U distribution coefficient (*K*_d_) value of 10^4^ between Fe(III) hydroxide and aqueous solution^32^,^33^ (taking the potentially lower seawater pH and higher atmospheric CO_2_ contents during the Archean relative to present conditions into consideration), Li and co-workers^31^ showed that the U concentration of 3.46 Ga seawater was at least two orders of magnitude lower (20–750 ppt) than that of modern seawater (3 ppb^34^), a result which according to Li and co-workers^31^ attests to anoxic atmospheric and ocean conditions at 3.46 Ga. Using the same parameters and 3.7 Ga for the decay correction, we calculated even lower U concentrations in the Isua seawater, with values ranging between 1.2 and 32 ppt (Fig. 3). The low redox potential of U(VI)/U(IV) couple makes U an element that is very sensitive to continental oxidation. We here emphasize, in conjunction with the positively fractionated Cr isotope data measured for the Isua BIF, that oxidative removal of U and Cr from the continental landmasses was apparently possible under very low oxygen levels of the 3.7 Ga atmosphere. The similarly elevated U/Th ratios in the MBC^31^ indicate that mobilization of U under low atmospheric oxygen levels may have persisted at least until 3.47 Ga.

Collectively, our data imply that oxidative processes were active under a very low oxygen atmosphere during the deposition of Isua sediments. Such oxidative processes, nevertheless, require the presence and action of ROS. Czaja and co-authors^23^ concluded that the δ^56^Fe distributions in Isua BIF reflect concentrations of <0.001% modern seawater O_2_ in the photic zone. Such low oxygen levels are well in line with independent proxies for atmospheric oxygen levels including the photochemical mass independent fractionation of S-isotopes^35^, which implies less than about 10^−3^ to 10^−5^ present atmospheric level (PAL) O_2_.

Large-scale Cr isotope fractionation preserved in marine sediments appears to be restricted to Neoproterozoic and younger marine sediments, implying that relatively high oxygen levels, of more than ~10^−3^ PAL may be needed to record fractionations on the order of several per mil. in marine sediments^12^,^14^. On the other hand, concentrations as low as 10^−5^–10^−4^ PAL appear sufficient to induce large Cr isotope fractionations in paleosols, and these continental signals can lead to mildly positive δ^53^Cr values in contemporaneous BIFs^11^. As an absolute lower limit, thermodynamic considerations suggest (Supplementary Figs 8,9) that Cr oxidation may be theoretically possible at O_2_ concentrations as low as 10^−20^ PAL at circumneutral pH, and that even if Cr oxidation were dependent on catalysis by Mn, it would have been possible at O_2_ concentrations below 10^−5^ PAL with pH above 4. The kinetics of Cr dissolution and oxidation including its microbial catalysis, however, need also be considered, and some models predict that extensive Cr oxidation and isotope fractionation take place at O_2_ concentrations of less than 0.1% PAL^36^.

The precise threshold O_2_ concentrations for the induction of Cr isotope fractionation remain uncertain, but we argue here that our data are consistent with the very low levels of oxygen or other ROS indicated by other proxies. If we estimate that weathering could generate a ~3% fractionation in Cr run-off^28^, then mass budget calculations using the average δ^53^Cr of 0.05% defined by the Isua BIFs would imply that less than only ~2% of the total Cr in the BIF would need to carry this signal. If 3.8–3.7 Ga rivers had total Cr concentrations on the order of 3 nmol l^−1^, comparable to Cr(III) concentrations in modern rivers^26^,^37^, only about 60 pmol l^−1^ of Cr would need to have seen a redox cycle. At these low concentrations, we emphasize that the redox active Cr pool need not necessarily be transported to the ocean as Cr(VI). In contrast, assuming that rivers during the early Archean were likely reducing in nature, such small amounts of redox active Cr could have been delivered to the oceans as soluble Cr(III) instead. Importantly, any trace of Cr that cycled through redox reactions on land would tend both to be heavy, and to mobilize into the contemporaneous run-off more readily than Cr weathered directly as Cr(III). Having reached the oceans, this fractionated Cr would have been stripped from seawater by Fe (oxy)hydroxides formed during the deposition of BIFs from low oxygen oceans.

Czaja and co-authors^23^ argued that iron deposition to form Isua BIF was likely the product of photoferrotrophic iron oxidation through anoxygenic photosynthesis rather than by oxygen produced through oxygenic photosynthesis. They further argued that if oxygenic photosynthesis had evolved and was active at this time, that cyanobacteria had not proliferated and that the oxygen produced must have been rapidly consumed leading to little Fe(III)-deposition compared to times later in the Archean. Similar arguments would apply to molecular oxygen produced directly through CO_2_ photodissociation^38^.

Hydrogen peroxide (H_2_O_2_) is another ROS, which could have led to iron oxidation in the Eoarchean. It can be generated through several processes and has been measured in a variety of different environments^39^ (and references therein), but Pecoits and co-workers^39^ consider H_2_O_2_ formed through atmospheric photochemical reactions as the only process that can generate significant fluxes. The formation of H_2_O_2_ in the ancient atmosphere is linked to initial photolysis of water vapour, or to the reaction of water vapour with an excited atomic oxygen^39^. Both OH and HO_2_ molecules, which are produced during these reactions, are precursors to H_2_O_2_ formation, and H_2_O_2_ may then be removed from the atmosphere by photolysis, reaction with OH, or by rainout^40^. Using conservative Fe(III) sedimentation rates predicted for submarine hydrothermal settings in the Eoarchean, Pecoits and co-workers^39^ argue that the flux of H_2_O_2_ produced in an Eoarchean atmosphere was likewise insufficient by several orders of magnitude to account for iron formation deposition.

In Paleoproteozoic BIFs, such as those of the Hamersley and Transvaal basins, concentrations of Fe^2+^_aq_ appear to have been drawn down leading to reservoir effects and iron isotope signatures with δ^56^Fe values close to the 0% of the contemporaneous seawater. In contrast, slightly positive δ^56^Fe values of the Isua BIF likely indicate very little Fe drawdown during partial oxidation^23^, implying that BIF deposition was not limited by Fe supply, and instead was either limited by oxidant availability or the activity of photoferrotrophs. Our data reveal a similar picture of low atmospheric oxygen concentrations, but we also demonstrate elemental cycling at relatively high redox potentials that requires at least the local presence of ROS. Such ROS may have been produced through the local activity of terrestrial oxygenic phototrophs^41^ if they had evolved at this early time, or through photochemical reactions^38^. Irrespective of their source, these ROS were likely consumed rapidly through reactions with abundantly available reductants and therefore failed to accumulate to high concentrations. Their presence, nevertheless, was sufficient to induce oxidative weathering detectable in Isua BIF. The reactive oxygen species recorded in Isua sediments may also have been sufficient to support the very early evolution of aerobic metabolisms, which are known to occur in extant bacteria at oxygen concentrations as low as 10^−8^ atm^42^.

## Methods

Individual cut-out mesobands were crushed in an agate shatter mill. Five gram aliquots of these powders were sent to ACME laboratories (Vancouver, Canada), where they were analyzed for major element compositions using ICP-OES (for analytical details, refer to http://acmelab.com/). Fifty mg of sample powders were dissolved in 7 ml Savillex™ beakers in a mixture of 2 ml concentrated HF and 3 ml of *aqua regia* (all Seastar™ acids) for 48 hours. After drying down the sample solutions, they were taken up with 1 ml concentrated HNO_3_ and diluted further to 10 ml with ultrapure water. This solution was analyzed for trace elements using a Perkin Elmer ELAN 6000 DRS ICP-MS at the Geological Survey of Denmark and Greenland (GEUS), using international standards^43^. A comparison of GEUS’ analytical results on some standards with published values are contained in Kalsbeek and Frei^44^.

Amounts of between 300 mg and 1 g of the powders were dissolved in the same way as those aliquots prepared for trace element analyses, with amounts of acids scaled accordingly. The samples were doped with an appropriate amount of a ^50^Cr-^54^Cr double spike. After 48 hours on a hotplate at 130 °C, the sample solutions were dried down. The samples first were re-dissolved in 6 N HCl and passed over a chromatographic column charged with 12 ml of an anion exchange resin, according to the procedure described in Frei *et al.*^14^ for the purpose of removing the iron matrix from the samples. After renewed evaporation of the Cr-cuts, the samples were then re-dissolved in 10 ml of 0.1 N HCl to which 3 drops of a 10% ammonium hydroxide solution and 3 drops of concentrated H_2_O_2_ were added to enable oxidation of Cr(III) to Cr(VI). After about 12 hours, the sample solution was then passed over PP extraction columns charged with 2 ml anion resin (Biorad ™AG-1 × 8, 100–200 mesh). Cr(VI), retained in the resin, was released by reduction to Cr(III) with the help of 10 ml 0.1 HNO_3_ doped with 3 drops of concentrated H_2_O_2_ into 12 ml Savillex™ Teflon beakers. After drying of this sample on a hotplate at 100 °C, the sample was re-dissolved in 200 μl of 6N HCl and, with the lid closed, was placed on a hotplate at 100 °C for 2–3 hours, during which the beaker was repeatedly tapped to prevent the solution in the beaker evaporating and fully condensing on the beaker’s surface. The sample was then diluted with 2 ml ultraclean water and passed over 2 ml of cation exchange resin (AG 50W-8, 200–400 mesh) charged PP columns. The extraction procedure followed a the slightly modified recipe of Bonnand *et al.*^45^ and Trinquier *et al.*^46^. With applying a two ion chromatographic column procedure we obtain highly pure Cr separates. Disturbing cations and anions are efficiently removed from the sample solutions during the oxidation-reduction step in the anion chromatographic separation, whereas remaining cations such as Ca^2+,^ Na^+^, and Mn^2+^ are removed in the respective elution procedure over the cation exchange column.

Chromium separates were measured on an IsotopX, model “Phoenix” TIMS, equipped with eight moveable Faraday collectors, in static mode. Loading and measuring procedures adhere to those reported by Frei and Polat^27^. We report Cr isotope compositions as δ^53^Cr = (^53^Cr/^52^Cr)_sample_/(^53^Cr/^52^Cr)_SRM 979_ − 1) × 1000, where SRM 979 denotes Standard Reference Material 979. We presently measure and externally reproduce the double spiked SRM 979 Cr standard at δ^53^Cr = 0.08 +/−0.05‰ (n = 263), with maintaining a ^52^Cr signal at 5E-12 Ampères (corresponding to a 500 mV beam intensity which we usually aim at for our sample analyses). A double spiked SRM 979 standard was irregularly interspersed in our analytical batches, in that way that a standard measurements was performed between every 3 to 4 sample measurements. The reported δ^53^Cr values and respective errors of the samples are calculated as the average of “n” repeated mass spectrometrical runs with their two standard deviations and include the correction for the offset of +0.08% for our SRM 979 from its accepted 0‰ δ^53^Cr value. A mass spectrometrical run consisted of 120 scans (divided in 12 blocks) with a signal integration period of ten seconds for each scan. Baselines were measured at the beginning of every second block over an integration period of 20 seconds each at +0.5 and −0.5 AMU from the Cr peaks. A typical mass spectrometrical run lasted ~40 minutes. Procedural Cr blanks were in the order of 2–4 ng and are insignificant relative to sample Cr amounts >350 ng (but typically ≫500 ng), i.e., they did not affect the measured Cr isotope composition of the samples.

During the time period where we measured our samples, we have interspersed analyses of the iron formation standard IF-G^47^. This standard was prepared from quartz-magnetite BIF of the IGB and is therefore a suitable material for comparative purposes with our mesoband data. δ^53^Cr values of 5 independently processed IF-G standards are listed in Supplementary Table 1 and plotted on Fig. 1a for reference. The average δ^53^Cr value of 0.03 +/−0.05% corresponds with the positively fractioned values of most of the mesobands analyzed herein.

## Additional Information

**How to cite this article**: Frei, R. *et al.* Oxidative elemental cycling under the low O_2_ Eoarchean atmosphere. *Sci. Rep.* **6**, 21058; doi: 10.1038/srep21058 (2016).

## Supplementary Material

Supplementary Table S1

Supplementary Table S2

Supplementary Information
