# Supplementary material for: Oxidative elemental cycling under the low O2 Eoarchean atmosphere
Source: Sci Rep. 2016 Feb 11;6:21058. doi: 10.1038/srep21058 (PMC4750038; doi:10.1038/srep21058)
Supplement: Supplementary Information [file srep21058-s3.pdf]

Supplementary Information

Oxidative elemental cycling under the low O_2 Eoarchean atmosphere

Robert Frei, Sean A. Crowe, Michael Bau, Ali Polat, David A. Fowle, Lasse N. Døssing

correspondence to: robertf@ign.ku.dk

This PDF file includes:

Supplementary information text
- Regional Geology
- Sample petrography
- Geochemical composition of the samples
- Stability fields for the Cr(III)/Cr(VI) and Mn(II)/Mn(III/IV) redox couples

Supplementary Figures 1 to 9
Supplementary Tables 1 and 2

.

Supplementary information text

Regional Geology

The 3.7-3.8 Ga Isua Greenstone Belt (IGB) of southern West Greenland is one of the oldest known successions of volcanic and sedimentary rocks. The IGB forms an arcuate belt comprising, among others, metabasaltic units and a major BIF unit deposited in water <200m[48], which have been subjected to amphibolite –facies metamorphism[49,50]. The similarity of geochemical compositions of the metavolcanic sequences in the ISB with modern arc-related boninites led Polat and co-workers[16] to propose an intra-oceanic subduction-like geodynamic setting for the formation of the basalts in the ISB.

The eastern portion of the IGB (Supplementary Fig. 1) is a region of low strain[51] and as such, metamorphic recrystallization is likely to be minimal there. This region includes three tectonic domains (Supplementary Fig. 1): A northwestern (NW) domain dominated by quartzo-feldspathic schists and garnet amphibolites, amphibolites and mica schists; A central domain (CD) with metamorphosed chert, banded iron formation and chlorite-amphibole schists (with relic pillow lava structures); and a southeast (SE) domain with essentially the same lithologies as the NW domain together with a sliver of carbonate and ultramafic schist. The BIF hosting CD is interpreted to be the region of least strain in the belt where primary depositional features are still recognizable[16,48]. The BIFs were subjected to metamorphic temperatures ranging from ~470 to 550°C in the CD[50].

Sample petrography

All samples, except 97-Isua-1 668.74M, were collected as surface outcrop samples within the central tectonic domain (CD; Supplementary Fig. 1) of the NE sector of the IGB. Sample localities are indicated on Figure S1 and Lat/Long coordinates are indicated in Supplementary Table 1. The samples are categorized into quartz-magnetite iron formation, mixed quartz-magnetite / magnesian iron formation, and magnesian iron formation, using the classification of Dymek and Klein[52] . This classification is based on the abundance of MgO which accounts for the presence of Mg,Fe-silicates. Iron formation samples with MgO < 2.5 wt% are termed quartz-magnetite iron formations, whereas MgO values >5.0 wt% are characteristic of magnesian iron formation. All samples were meticulously inspected for signs of secondary alteration, particularly with respect to cross-cutting veins and with respect to secondary accessory and refractory phases (such as monazite, apatite and allanite) which potentially could contribute to the bulk U contents of the mesobands. For this, we have used the back scatter electron imaging feature of the JEOL Superprobe at the Department of Geoscience and Natural Resource Management (University of Copenhagen). The BIF samples used for the chromium isotope analyses were devoid of such accessory phases and other apparent secondary features. Photographs of hand specimen samples of the studied BIF are presented in Supplementary Fig.2.

In addition, two garnet micaschists, two quartzo-feldspathic schists and four metabasalts from the CD were analyzed for their Cr concentrations and Cr isotope compositions (Supplementary Table 1). These samples, described in detail by Polat and co-workers[16-18], are tightly genetically and depositionally related to the BIF samples studied herein. Petrographic and isotopic studies suggested that the Isua felsic and garnet-mica schists were derived from proximal juvenile volcanogenic sources[53]. Geochemical data from these rocks are consistent with an arc signature and a heterogeneous source area[18]. The geochemical characteristics of the volcanic rocks within the CD

are comparable to those of Phanerozoic boninites[16] and suggest an intra-oceanic subduction zone-like geodynamic setting for the formation of these basalts. In summary, the structural and geochemical characteristics of volcanic rocks and volcanogenic sediments are consistent with a convergent margin-like, supra-subduction-type geodynamic setting for the Isua greenstone belt[16-18].

Individual mesobands of the 7 BIF samples studied herein, including a drill core with hematite facies BIF from beneath the inland ice sheet (sample 97-Isua-1 668.74M, drilled in 1997 by the Krylolitselskab Øresund consortium to investigate the western extension of the iron formation beneath the ice sheet) were cut from one-centimeter thick rock slices with a diamond blade saw and individually powdered in an agate mortar. Aliquots of these powders were used for major and trace element analyses, and for Cr isotope analyses (see Methods section in main text).

Quartz-magnetite BIF (Supplementary Fig. 3 A,B) is primarily composed of quartz, magnetite, and of subordinate (<1% modal) Fe-Mg amphiboles (grunerite-cummingtonite) and, very seldom dolomite. Accessories are hematite (specularite) and apatite.

Mixed quartz-magnetite / magnesian BIF (Supplementary Fig. 3 C,D) is compositionally very similar to quartz magnetite BIF, but with a higher abundance (>1% modal) of Fe-Mg amphiboles.

Magnesian BIF samples (Supplementary Fig. 3 E,F) have up to 20% modal amphibole (grunerite, cummingtonite). Amphiboles show a clear overgrowth texture with a preferred orientation of the laths, which in the illustrated sample is at angle with the primary bedding indicated by magnetite and quartz.

Geochemical composition of the samples

Supplementary Table 1 lists the major and trace elemental composition, and the $\delta^{53}Cr$ values of mesobands from the 7 studied BIF samples and the IF-G BIF standard[47] from Isua.

Eu, Gd, Ce, Pr and La anomalies, parameters which are used in the characterization of marine chemical precipitates[4] , were calculated as anomalies relative to Average Post-Archaean Australian Shale (PAAS[54]), in the form of REE_{SN}/REE^*_{SN}, with $Eu^*_{SN} = 0.67Sm_{SN} + 0.33Tb_{SN}$, $Gd^*_{SN} = 0.33Sm_{SN} + 0.67Tb_{SN}$, $Ce^*_{SN} = 0.5La_{SN} + 0.5Pr_{SN}$, $Pr^*_{SN} = 0.5Ce_{SN} + 0.5Nd_{SN}$, and $La^*_{SN} = 3Pr_{SN} – 2Nd_{SN}$ (SN = shale normalized).

Major and trace element concentrations in the 57 mesobands analyzed herein (Supplementary Table 1) reflect the bimodal mineralogical composition of the sample set, i.e. silica bands (SiO_2 > 40%) and magnetite bands (Fe_2O_3 > 40%), with mesobands characterized by different proportions of magnetite and silica (quartz) falling in between the two limits. MnO concentration are generally very low, exhibiting a range between <0.01 and 0.32%. The lowest concentrations are in quartz-magnetite BIF samples, whereas those BIFs with higher MgO concentrations also have slightly elevated MnO concentrations. The detrital contamination of the sediments can be assessed by Al_2O_3 concentrations, or by other lithogenic immobile element concentrations such as Zr, Sc, Th, Ti etc. Al_2O_3 concentrations remain below 1.36%, but typically below 0.5%, in the mesobands analyzed, and they are lowest is quartz-magnetite BIF samples (average of 0.1%). These values are compatible with Zr concentrations remaining <13 ppm, values that are comparable with chemically pure Neoarchean iron formation, such as the Temagami iron formation (Canada)[55] or Archean to

Paleoproterozoic BIFs from a number of worldwide locations studied in detail for their REE + Y compositions by Planavsky and co-workers[56].

We here want to particularly discuss the REE+Y (REY) compositions and the coupled Cr-U enrichments of the IGB samples, in the light of a seawater paelo-redox characterization and its potential link to environmental conditions on land at this time.

REY patterns

REY patterns of the samples are plotted in Supplementary Fig. 4 (quartz-magnetite BIF, mixed quartz-magnetite / magnesian BIF, magnesian BIF and hematite BIF. REY patterns for 5 replicates of the iron formation standard IF-G[47] are plotted in Supplementary Fig. 5. The REY distributions of the mesobands from the samples studied herein are very similar to those of other Archean iron formations, which are low in or free from clastic detritus. The general shape of the REY patterns and details such as positive Gd and positive Y/Ho anomalies are similar to the REY distribution in modern seawater (except the redox-sensitive Ce and Eu), supporting the assertion that the IGB BIF samples represent marine chemical sediments that can be used as archives for recording the evolution of the Early Earth’s atmosphere-hydrosphere system.
Magnesian BIF samples (IGB-IF32,33) exhibit variations in the $(Nd/Yb)_{SN}$ ratio (indicator for the degree of LREE depletion), with generally less depleted LREE compared to quartz-magnetite- and mixed quartz-magnetite / magnesian BIF, but there is no systematic correlation between MgO and this parameter. $(Nd/Yb)_{SN}$ values of quartz-magnetite and mixed quartz-magnetite / magnesian BIF samples are low (range between 0.03 – 0.72) and correspond, for example, with those reported for magnetite and silica bands for the ca. 2.7 Ga Temagami iron formation by Bau and Alexander[55].

Our study however does not reveal the systematic difference in more LREE depleted pattern of silica bands relative to magnetite bands as reported by these authors. However, in some samples (e.g., IGB-IF31 or 97-ISUA-1 668.74M) there is quite some variability in the degree of LREE depletion amongst the different bands analyzed, which seem to somehow negatively correlate with Al_2O_3 contents (not shown) and consequently cannot therefore be explained by degree of detrital shale contamination. A possible explanation for this could be that the continental landmass at Isua time (~3.7-3.8 Ga) was characterized by a reworked, initially basaltic Hadean protocrust, which was for example emphasized by the Pb isotope studies of Kamber and co-workers[57].

None of the BIF samples studied contain statistically significant negative Ce/Ce^*_{SN} anomalies (Supplementary Fig. 5). True negative Ce/Ce^*_{SN} anomalies have Ce/Ce^*_{SN} and Pr/Pr^*_{SN} values less than and greater than unity, respectively. This approach, described by Bau and Dulski[4], discriminates between positive La and true negative Ce anomalies. A few positive Ce anomalies are constrained to samples IGB-IF31 and 33 (a magnesian BIF and and mixed quartz-magnetite / magnesian BIF, respectively), but are absent in quartz-magnetite BIFs. The general absence of negative Ce anomalies in the BIFs studied indicate that the waters from which they were deposited were likely suboxic or anoxic and that the fO_2 was not sufficient to oxidize Ce(III). Whether or not the variations in LREE depletion of some samples and the observation of slightly positive Ce anomalies are connected to the former presence of a sporadic stratified water column in the Isua basin, in which Mn cycling and reductive dissolution of Mn-Fe (oxyhyd)oxides and Ce(IV) in a suboxic zone could have variably increased the LREE relative to HREE ratio of the overlying water column as proposed by Sholkovitz and co-workers[58] cannot be assessed in detail. However, the presence, at least sporadically, of a stratified water column in the Isua basin is supported by the Cr isotope data presented herein. Last not but least, the Ce/Ce^*_{SN} vs Pr/Pr^*_{SN} relationships displayed by the BIF samples indicate an analytically significant positive La anomaly, a feature which has

been interpreted to derive from enhanced river water contribution[59] to basin waters. Strong Eu anomalies are characteristic of the IGB BIFs. These are a clear indication that high-temperature hydrothermal solutions similar to modern black-smoker-type fluids were an important source of the REE+Y inventory of the Isua basin. Eu/Eu^*_{SN} values of all mesobands are rather homogenous defining a value of 3.1 +/- 0.4. We however note small interband variability in each of the studied samples, and this renders it unlikely that adjacent magnetite and silica bands in these BIFs formed by diagenetic separation of an originally homogenous Fe-oxide+silica precipitate into discrete Fe-oxide and silica bands since the Eu-REE decoupling requires temperatures above at least 200°C, as discussed in the case of the Temagami iron formation by Bau and Alexander[55]. A strong hydrothermal input into the Isua basin is compatible with the proposed geodynamic scenario of a convergent margin setting in which the emplacement of subaqueous basalts with boninitic and picritic geochemical affinities, associated with the BIF in the IGB, has been envisaged by Polat and co-workers[16,17]. Similar Pb isotopic compositions in pillow basalts and BIFs[18] are consistent with a common source of Pb for both supracrustal rocks. Iron and Pb were probably scavenged from the oceanic crust and were re-deposited on the ocean floor by hydrothermal fluids, suggesting that high-temperature hydrothermal alteration of early Archean oceanic crust played an important role in the deposition of the Isua BIFs.

U and Cr relationships

Uranium concentration in IF has been advocated by Partin and co-workers[22] as a useful tracer of soluble U(VI) in seawater, and indirectly, as a tracer for oxidative continental weathering. The basis behind this proposal lies in the fact that the geochemical cycles of U and Fe are closely linked, with the U(VI)/U(IV) and Fe(III)/Fe(II) redox couples being similar in reduction potential. Most U

in IF is likely to have been derived directly from seawater by adsorption and co-precipitation processes, and it is therefore that we here propose a strong correlation to be expected with Cr. Similar to U, we here propose that the predominant source of Cr is riverine runoff [26], which in turn reflects oxidative weathering and the conversion of soil/rock-hosted Cr(III) to soluble Cr(VI)[11,14,25-27]. We here use U/Th ratios in the BIF mesobands, relative to the U/Th ratio of 0.2564 defined by average Archean crust[20], to quantify the U enrichment. The U/Th ratio is equally distributed and cannot be statistically distinguished between the different types of BIF studied (Supplementary Table 1). An average U/Th ratio of 0.68 +/- 0.28 (1σ; n=57; Fig.1B) is calculated for all samples (excluding the weathered sample Isua- 97-1 668.74M) and statistically discriminates towards a higher value compared to the Archean average crustal value of 0.25[20] (Supplementary Table 2). U concentrations are somehow positively correlated (R^2=0.53) with Fe_2O_3/SiO_2 (Fig. 2B), which indicates the affinity of U adsorption onto the Fe(oxhydr)oxide precursor in the BIFs. This observation is consistent with the results of Partin and co-workers[22] who found that U is more abundant in laser-ablation data of iron oxides of various IF which they used to argue against homogeneous nucleation of reduced U mineral phases in the water column and to support the contention that adsorption or reduction at the mineral surface were the principal stripping mechanisms of U from the seawater. A slightly better correlation (R^2~0.73) is also depicted by Cr concentrations with Fe_2O_3/SiO_2 (Fig. 2A), and together with the positive correlation between Cr and U concentrations (R^2=0.74; Fig. S6) this not only indicates a likely common (continental) source for the two elements, but also a common stripping mechanism of U and Cr into the (oxhydr)oxide precursor in the BIFs.

Based on the distribution of U in organic-rich marine sediments deposited throughout Earth's history, Partin and co-workers[60] suggested that the size of the marine U reservoir tracks the redox evolution of the atmosphere and ocean system, since the only source of dissolved U to the ocean is

via oxidative continental weathering and riverine supply. Irrespective of whether by equilibrium U(VI) surface complexation to ferric (oxyhydr)oxide particles or by rapid reduction of U(VI) by Fe(II), similar to Cr(VI), at the iron (oxyhydr)oxide surface, we here propose that the net U and Cr enrichment observed in the BIF samples from the ISB, together with their preferential affinity with Fe_2O_3-rich mesobands, track the U and Cr concentrations in the overlying water column and provide a link to oxidative removal of these elements on land.

Stability fields for the Cr(III)/Cr(VI) and Mn(II)/Mn(III/IV) redox couples

The speciation of Cr and Mn (Supplementary Figs. 8 and 9) was calculated as a function of oxygen fugacity and pH using Geochemists Workbench (Version 10, using the LLNL thermo.dat database). For simplicity, Supplementary Fig. 9 only delineates the redox boundaries for Cr (red) and Mn (blue). Cr activities were set at 10^{-8}, whereas Mn activities were set at 10^{-5} - activities that might be expected in a circumneutral low oxygen weathering environment or microoxic ocean. All calculations were conducted at 25°C. Overall, our calculations show that the oxidation of Cr(III) to Cr(VI) is thermodynamically possible at oxygen concentrations well below 10^{-20} atm at pH conditions above about 7 (Supplementary Fig. 9). If oxidized Mn species are required to initiate Cr redox cycling, a pH above about 4 would be necessary at O_2 levels below about 10^{-5} atm (Supplementary Fig. 8). Photochemical processes are thought capable of producing O_2 at Earth's surface up to a maximum of about 3 x 10^{-6} bar[38], and at pH values of between 6 and 8, this is more than enough to initiate oxidative Cr and Mn cycling.

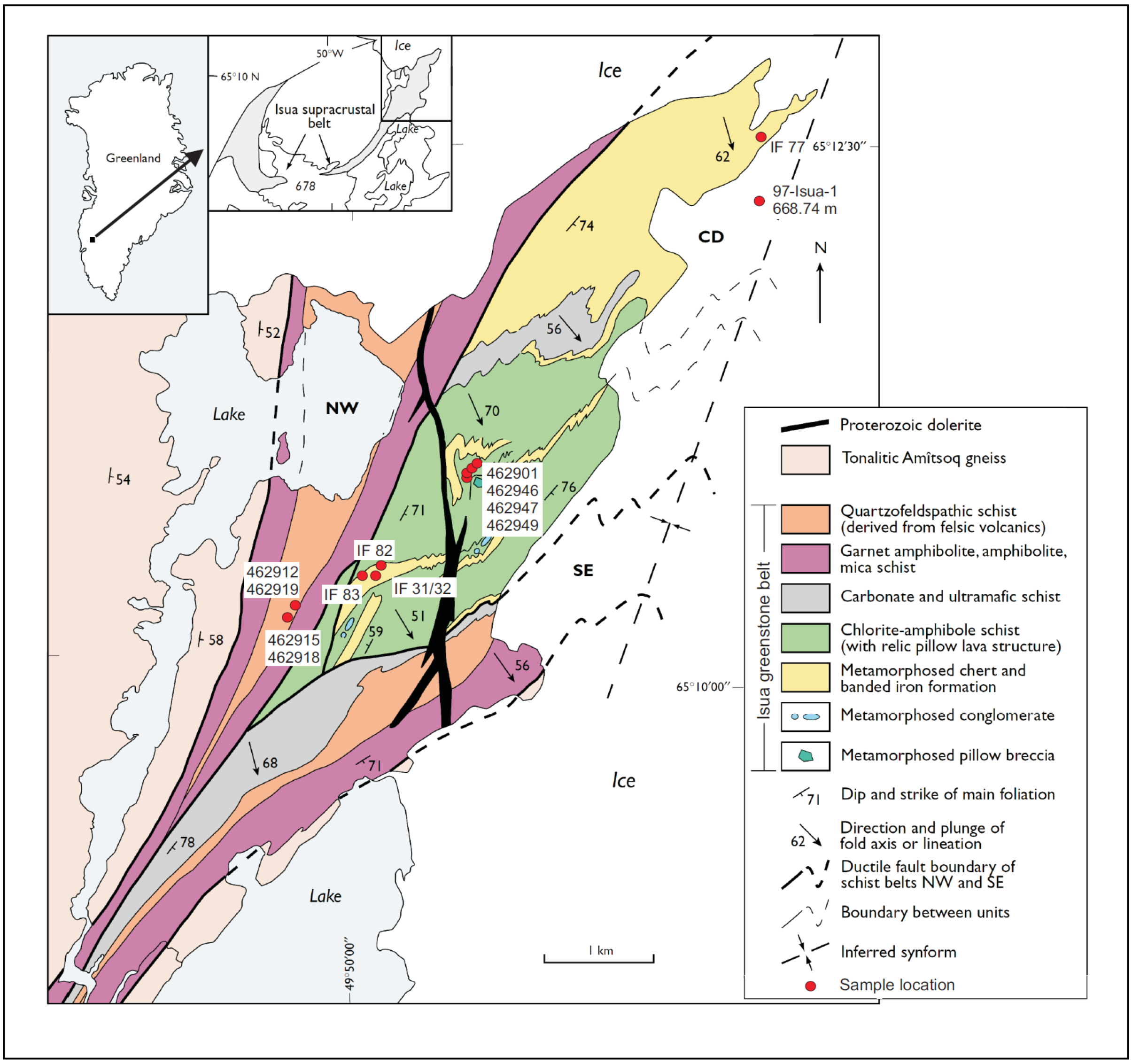

Supplementary Figure 1 | Simplified geological map of the eastern portion of the Isua Greenstone Belt. The central tectonic domain (CD) is a region of low strain and as such, metamorphic recrystallisation is likely to be minimal there. Locations of BIF samples studied herein are marked with a red-filled circle, together with the locations of depositionally and genetically associated metabasalts and clastic metasediments described in detail by Polat and co-workers[16-18]. Modified after Appel[51].

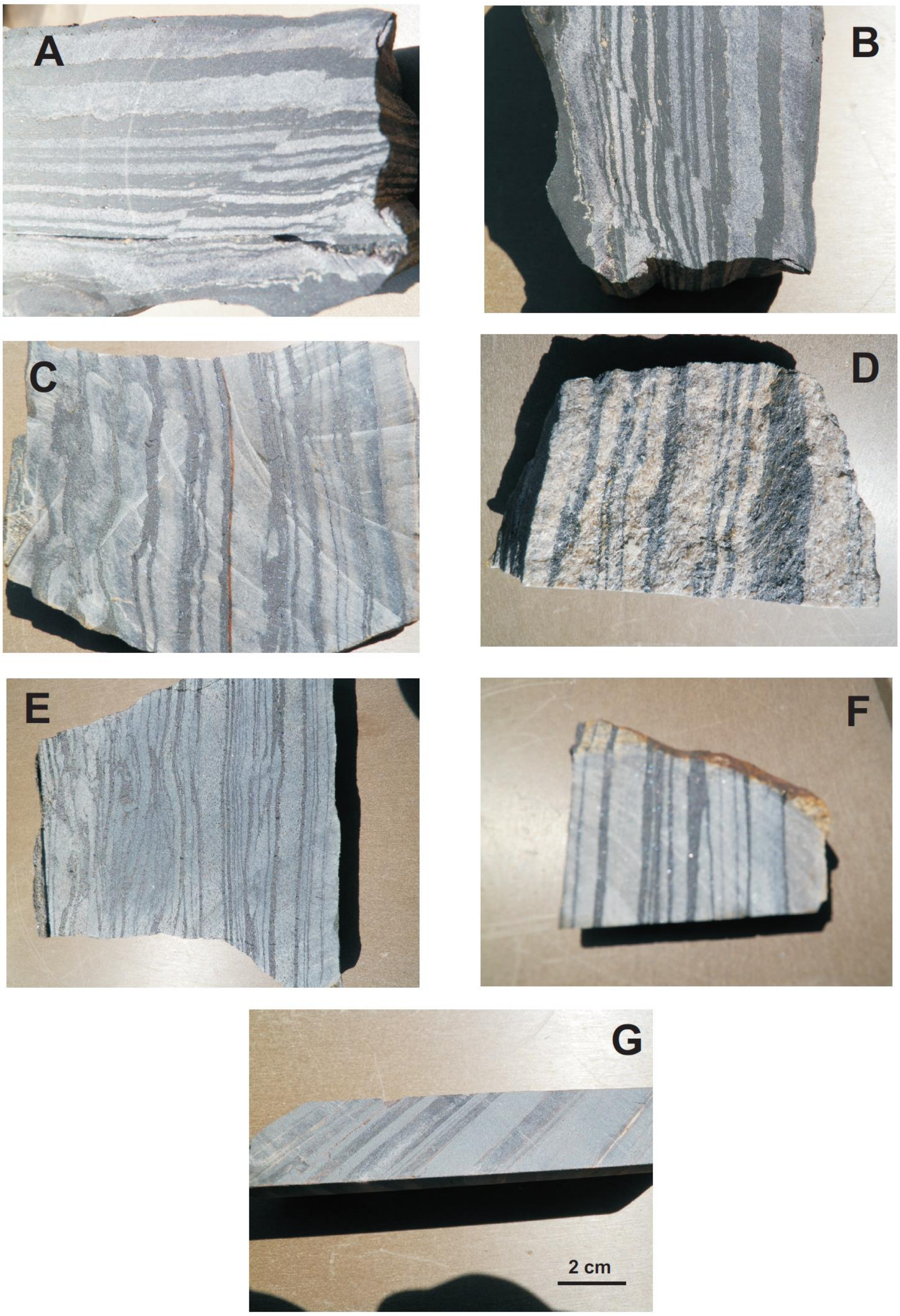

Supplementary Figure 2 | Photographs of handspecimen of studied BIF samples from Isua. A: sample IGB-IF73; B: sample IGB-IF 77; C: sample IGB-IF33; D: sample IGB-IF-32; E: sample IGB-IF82, F: sample IGB-IF31; and G: sample 97-Isua-1 668.74M (drill core). Scale bar in “G” applies to all panels.

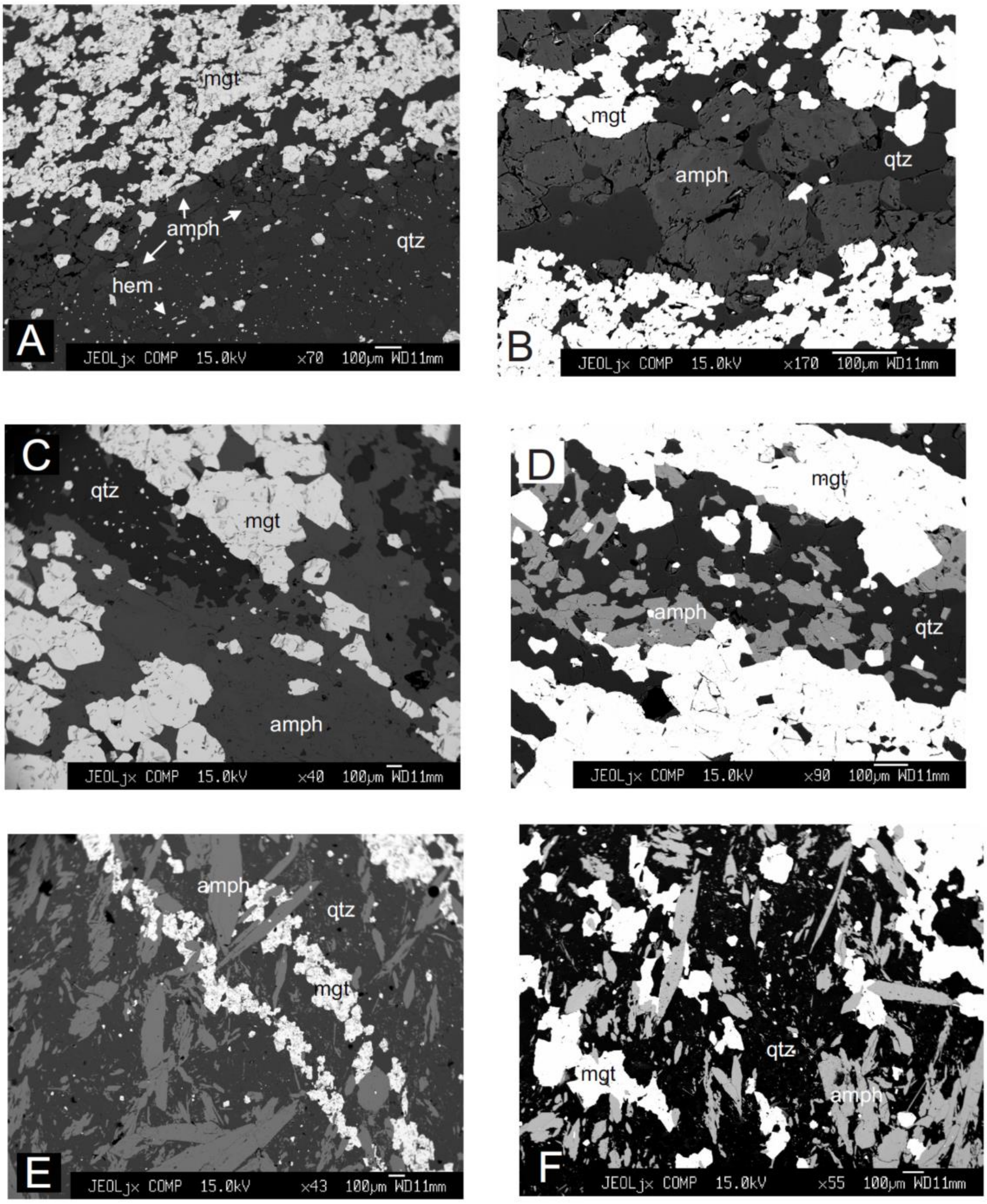

Supplementary Figure 3 | Electron microscope backscatter images of the three main types of BIF. Quartz-magnetite BIF (A,B), mixed quartz-magnetite / magnesian BIF (C,D) and magnesian BIF (E,F). mgt=magnetite; qtz=quartz; amp=amphibole; hem=hematite.

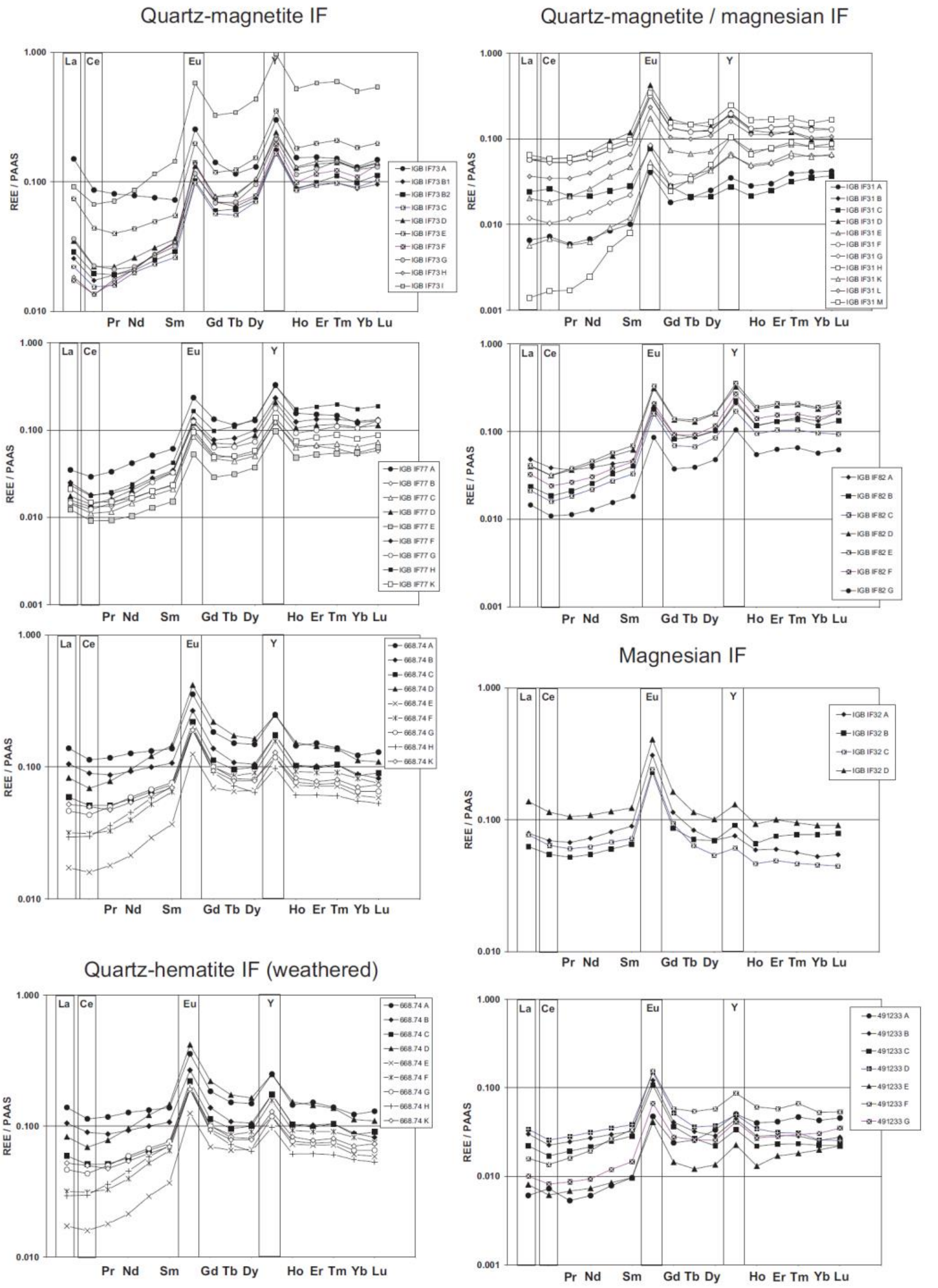

Supplementary Figure 4 | Rare Earth Element and Yttrium patterns in BIFs from Isua. Average Post-Archaean Australian Shale (PAAS[54]-normalized Rare Earth Element (REE) + yttrium (Y) patterns of mesobands from the three types of iron formation (IF; quartz-magnetite IF; quartz-magnetite/magnesian IF; magnesian IF) from Isua. The REE + Y distributions of the mesobands from the samples studied herein are very similar to those of other Archean iron formations which are low in or free from clastic detritus. The general shape of the REE+Y patterns and details such as positive Gd and positive Y/Ho anomalies are similar to the REE+Y distribution in modern seawater (except the redox-sensitive Ce and Eu), supporting the assertion that the IGB BIF samples represent marine chemical sediments that can be used as archives that record the evolution of the Early Earth’s atmosphere-hydrosphere system.

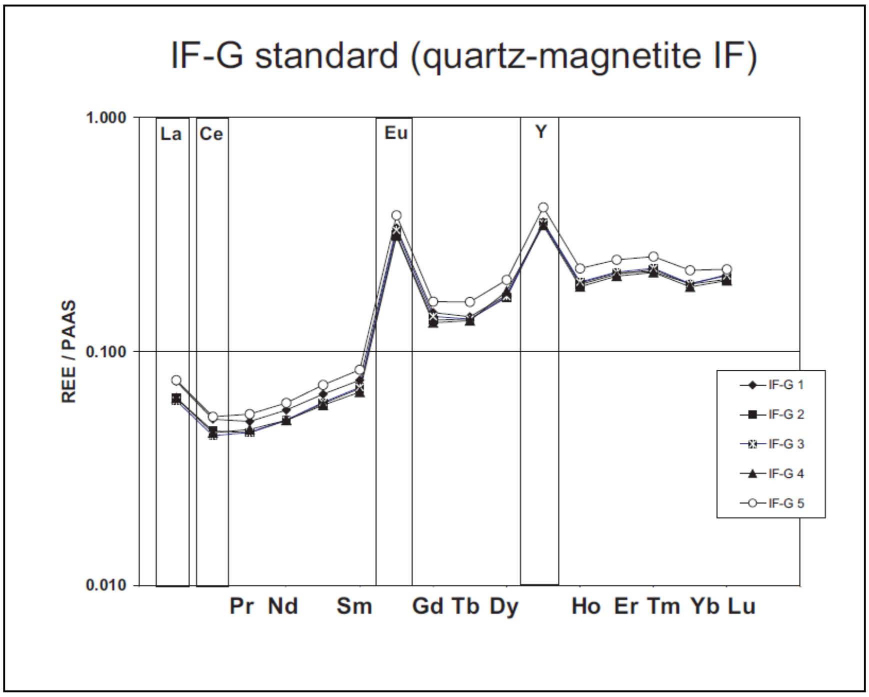

Supplementary Figure 5 | Rare Earth Element and Yttrium patterns of standard IF-G from Isua. Average Post-Archaean Australian Shale (PAAS[54]-normalized Rare Earth Element (REE) + yttrium (Y) pattern of 5 replicates of the IF-G iron formation standard from Isua.

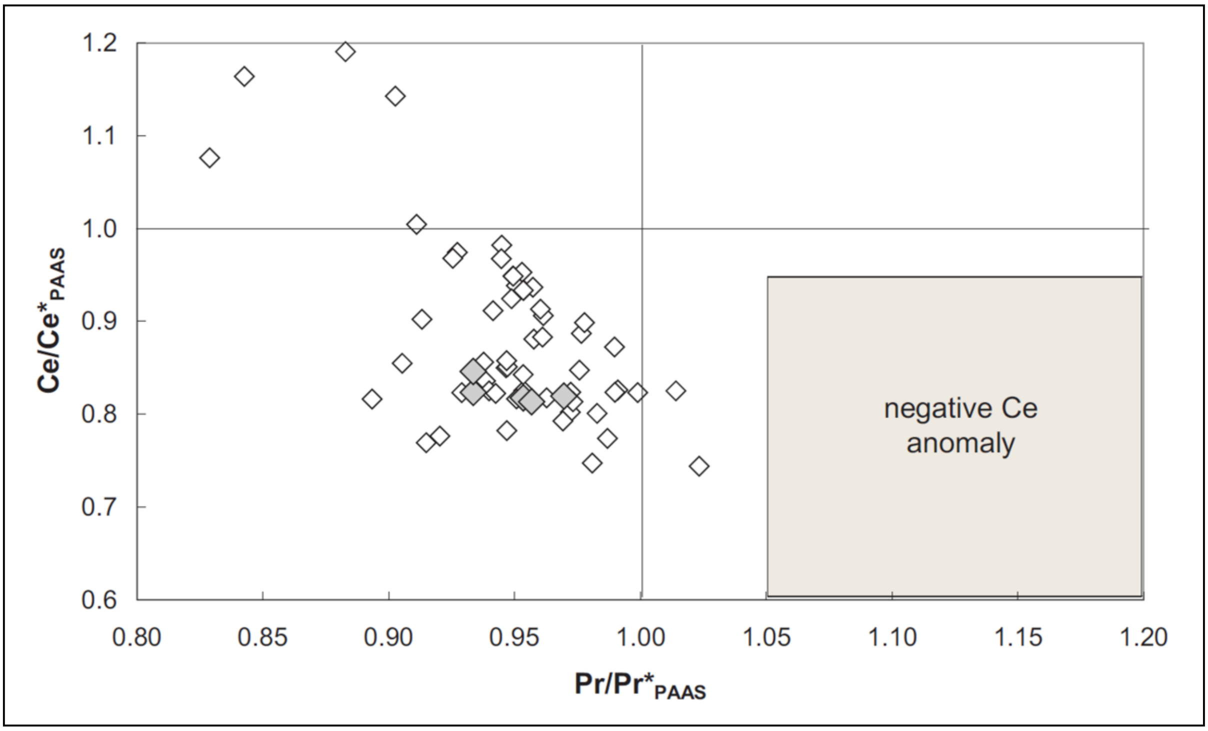

Supplementary Figure 6 | Praesodymium (Pr) vs. cerium (Ce) anomaly diagram of BIF mesobands from Isua. Data from the IF-G standard are plotted with grey-filled diamonds. True negative Ce/Ce^*_{PAAS} anomalies have Ce/Ce^*_{PAAS} and Pr/Pr^*_{PAAS} (PAAS= Average Post-Archaean Australian Shale[54]) values less than and greater than unity, respectively (grey filled area). The general absence of negative Ce anomalies in the BIFs studied indicate that the waters from which they were deposited were likely suboxic or anoxic and that the *f*O2 was not sufficient to oxidize Ce(III).

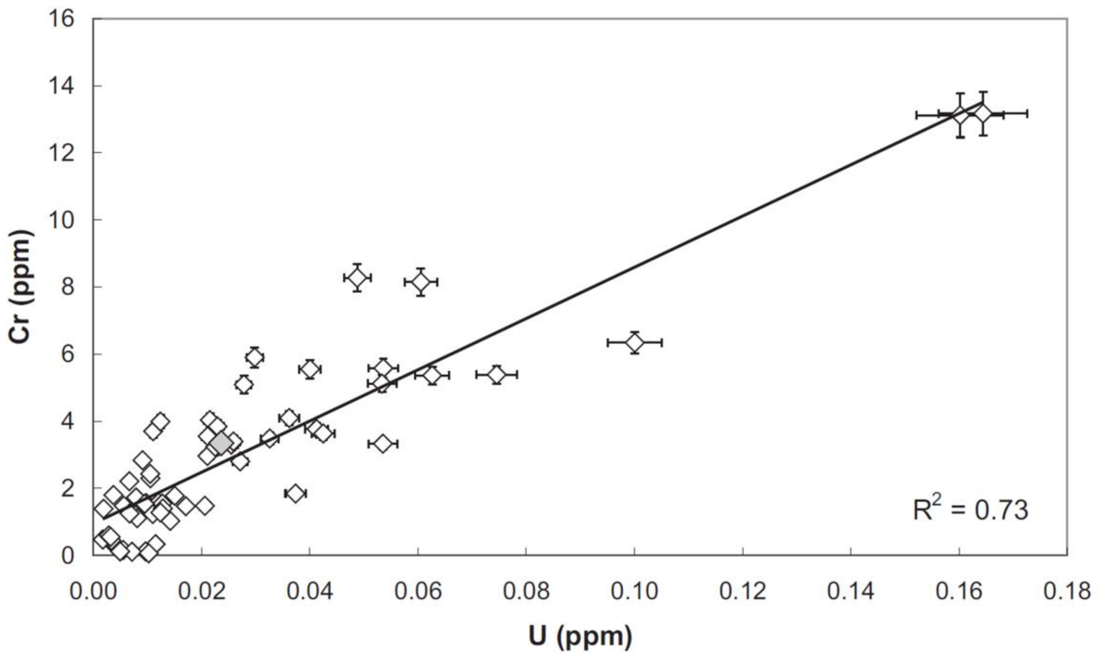

Supplementary Figure 7 | Uranium (U) *vs.* chromium (Cr) concentration diagram with data from individual mesobands of BIF samples from Isua. The fairly well-defined positive correlation between U and Cr concentrations (R^2=0.73) indicates a likely[31] common (continental) source for the two elements, but also a common stripping mechanism of U and Cr into the Fe(oxhydr)oxide precursor in the BIFs. The grey-filled symbol marks the average composition of the IF-G BIF standard.

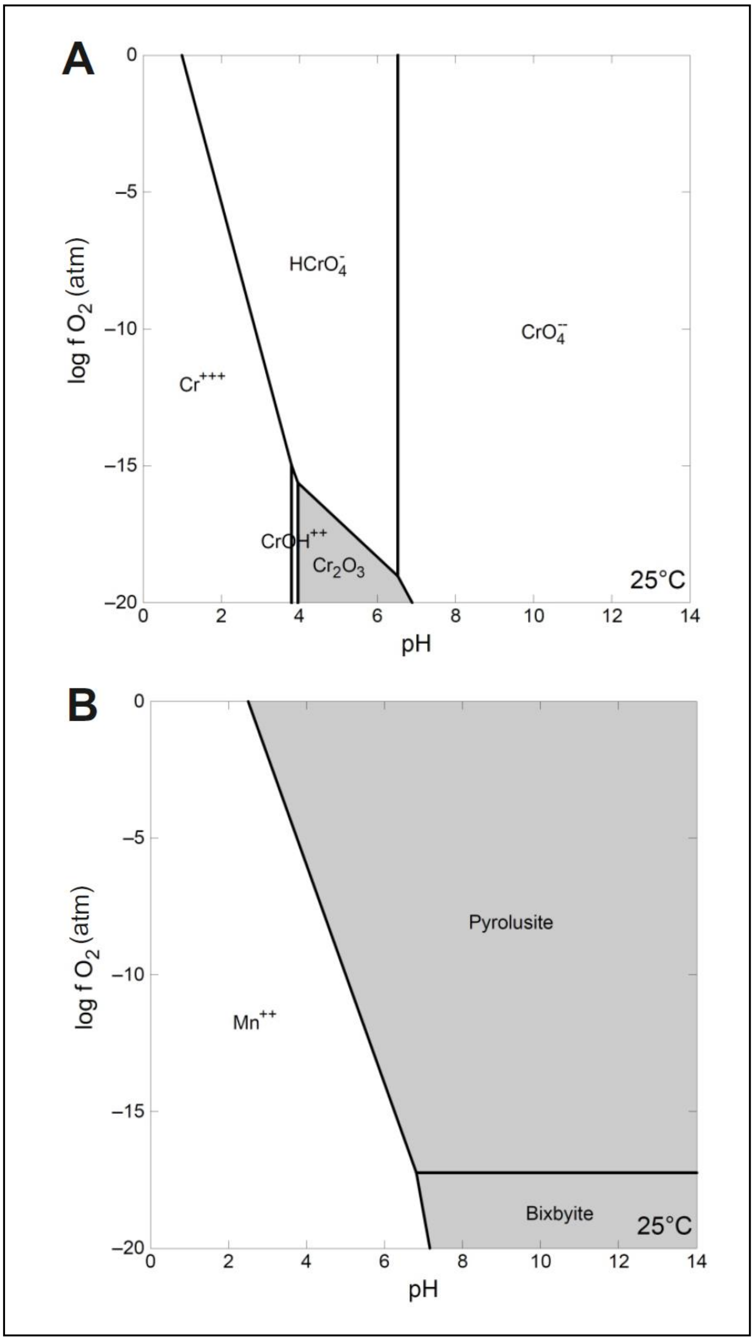

Supplementary Figure 8 | Speciation diagrams for the Cr and Mn redox couples. Oxidation of Cr(III) to Cr(VI) is thermodynamically possible at oxygen concentrations well below 10^{-20} atm at pH conditions above about 7 (A). If oxidized Mn species are required to initiate Cr redox cycling, a pH above about 4 would be necessary at O_2 levels below about 10^{-5} atm (B), activities that might be expected in a circumneutral low oxygen weathering environment or microoxic ocean.

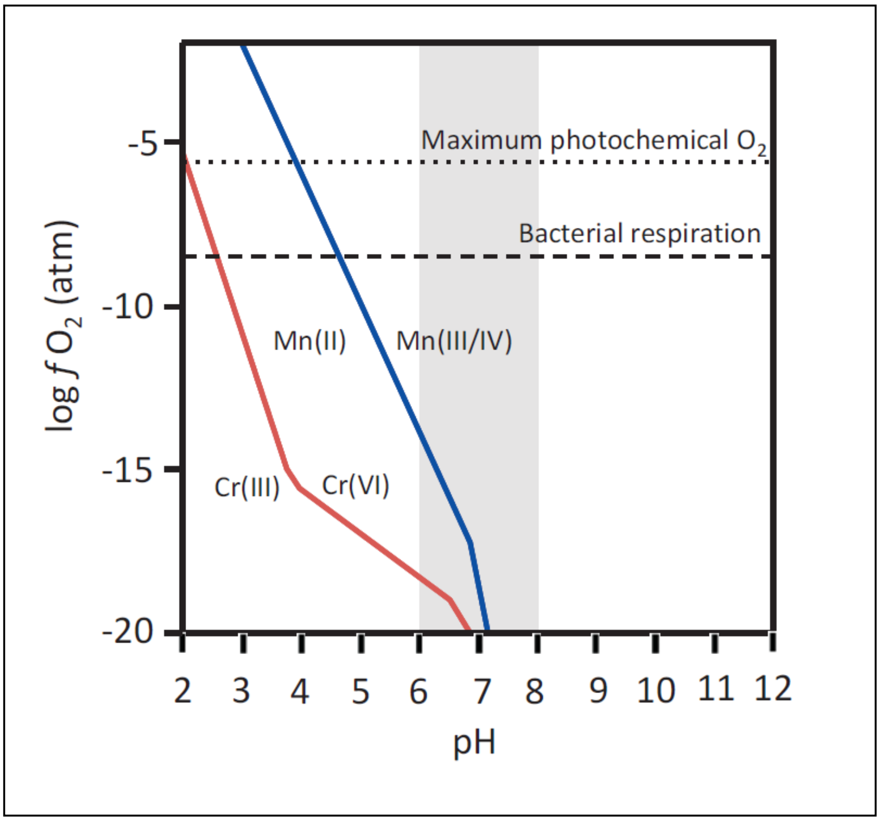

Supplementary Figure 9 | Stability fields for the Cr(III)/Cr(VI) and Mn(II)/Mn(III/IV) redox couples as a function of pH and log fO_2. The diagram shows that even at very low O_2 fugacity, both Cr(VI) and Mn(III/IV) are thermodynamically favoured under circumneutral pH conditions (6-8; grey-filled area). Atmospheric photochemistry is able to produce up to 3 x 10^{-6} bar of O_2 at the Earth's surface (dotted line)[38]. This is sufficient to initiate Mn and Cr redox cycling, even under circumneutral pH and is therefore also sufficient to generate, at least locally, an isotopic fractionation of Cr in the early Archean weathering environment. Also shown for reference are oxygen concentrations demonstrably accessible to aerobic bacteria[42].

References

48 Fedo, C. M., Myers, J. S. & Appel, P. W. U. Depositional setting and paleogeographic implications of earth's oldest supracrustal rocks, the >3.7 Ga Isua Greenstone Belt, West Greenland. *Sedimentary Geology* **141**, 61-77, (2001).

49 Boak, J. L. & Dymek, R. F. Metamorphism of the ca. 3800 Ma supracrustal rocks at Isua, West Greenland: implications for early Archean crustal evolution. *Earth Planet. Sci. Lett.* **59**, 155-176, (1982).

50 Rollinson, H. Metamorphic history suggested by by garnet-growth chronolgies in the Isua Greenstone Belt, West Greenland. *Precambrian Res.* **126**, 181-196, (2003).

51 Appel, P. W. U., Fedo, C. M., Moorbath, S. & Myers, J. S. Recognizable primary volcanic and sedimentary features in a low strain domain of the highly deformed, oldest known (3.7 – 3.8 Gry) Greenstone belt, Isua, West Greenland. *Terra Nova* **10**, 57-62, (1998).

52 Dymek, R. F. & Klein, C. Chemistry, Petrology and Origin of Banded Iron-Formation Lithologies from the 3800-Ma Isua Supracrustal Belt, West Greenland. *Precambrian Res.* **39**, 247-302, (1988).

53 Jacobsen, S. B. & Dymek, R. F. Nd and Sr isotope systematics of clastic metasediments from Isua, west Greenland: Identification of pre-3.8 Ga differentiated crustal components. *Journal of Geophysical Research.* **93**, (1988).

54 Taylor, S. R. & McLennan, S. M. *The continental crust: Its composition and evolution*. (Blackwell, 1985).

55 Bau, M. & Alexander, B. W. Distribution of high field strength elements (Y, Zr, REE, Hf, Ta, Th, U) in adjacent magnetite and chert bands and in reference standards FeR-3 and FeR-4 from the Temagami iron-formation, Canada, and the redox level of the Neoarchean ocean. *Precambrian Res.* **174**, 337-346, (2009).

56 Planavsky, N. *et al.* Rare Earth Element and yttrium compositions of Archean and Paleoproterozoic Fe formations revisited: New perspectives on the significance and mechanisms of deposition. *Geochim. Cosmochim. Acta* **74**, 6387-6405, (2010).

57 Kamber, B. S., Collerson, K. D., Moorbath, S. & Whitehouse, M. J. Inheritance of early Archaean Pb-isotope variability from long-lived Hadean protocrust. *Contrib. Mineral. Petrol.* **145**, 25-46, (2003).

58 Sholkovitz, E. R., Shaw, T. J. & Schneider, D. L. The Geochemistry of Rare-Earth Elements in the Seasonally Anoxic Water Column and Porewaters of Chesapeake Bay. *Geochim. Cosmochim. Acta* **56**, 3389-3402, (1992).

59 Elderfield, H., Upstillgoddard, R. & Sholkovitz, E. R. The Rare-Earth Elements in Rivers, Estuaries, and Coastal Seas and Their Significance to the Composition of Ocean Waters. *Geochim. Cosmochim. Acta* **54**, 971-991, (1990).

60 Partin, C. A. *et al.* Large-scale fluctuations in Precambrian atmospheric and oceanic oxygen levels from the record of U in shales. *Earth Planet. Sci. Lett.* **369**, 284-293, (2013).

Supplementary Table 1 | Major and selected trace elements, important element ratios and δ^{53}Cr values of BIF, metabasalt and metasediment samples from the IGB, West Greenland

Sample	Type	SiO_2	Al_2O_3	Fe_2O_3	MnO	MgO	CaO	Na_2O	K_2O	TiO_2	P_2O_5	δ^{53}Cr	+/- 2σ	n*
		wt%	wt%	wt%	wt%	wt%	wt%	wt%	wt%	wt%	wt%	‰		
N 65° 10.354' W 49° 49.277'														
IGB IF33A	Magnesian IF	77.31	0.08	16.11	5.92	5.92	0.04	<0.01	<0.01	<0.01	<0.01	0.06	0.06	10
IGB IF33B	Magnesian IF	51.06	0.22	45.11	3.90	3.90	0.05	<0.01	<0.01	<0.01	0.04	0.31	0.06	20
IGB IF33C	Magnesian IF	63.37	0.15	30.43	5.93	5.93	0.06	<0.01	<0.01	<0.01	0.04	0.09	0.06	20
IGB IF33D	Magnesian IF	50.20	0.23	46.34	4.14	4.14	0.06	<0.01	<0.01	<0.01	0.05	0.42	0.06	20
IGB IF33E	Magnesian IF	72.97	0.09	19.79	7.04	7.04	0.05	<0.01	<0.01	<0.01	0.03	0.03	0.06	10
IGB IF33F	Magnesian IF	33.77	0.20	63.22	0.07	2.65	0.05	<0.01	<0.01	<0.01	0.04	0.09	0.06	10
IGB IF33G	Magnesian IF	46.17	0.21	47.97	0.14	5.44	0.05	<0.01	<0.01	<0.01	0.03	0.04	0.06	10
N 65° 10.325' W 49° 49.081'														
IGB IF32 A	Magnesian IF	37.90	0.63	60.10	0.06	2.27	0.12	0.04	<0.01	0.02	0.03	0.07	0.06	20
IGB IF32 B	Magnesian IF	63.39	0.25	31.18	0.12	4.63	0.09	<0.01	<0.01	<0.01	0.06	0.15	0.05	8
IGB IF32 C	Magnesian IF	48.27	0.35	48.35	0.09	3.29	0.08	<0.01	<0.01	0.01	0.08	0.07	0.04	20
IGB IF32 D	Magnesian IF	54.74	1.36	39.47	0.07	3.76	0.09	<0.01	0.02	0.02	0.08	0.09	0.04	20
IGB IF82A	Quartz-magnetite IF / Magnesian IF	41.61	0.16	50.26	3.42	3.42	3.70	0.03	<0.01	<0.01	0.11	-0.04	0.06	16
IGB IF82B	Quartz-magnetite IF / Magnesian IF	54.56	0.09	42.66	1.36	1.36	1.49	<0.01	<0.01	<0.01	0.06	-0.04	0.06	10
IGB IF82C	Quartz-magnetite IF / Magnesian IF	59.98	0.08	37.55	1.35	1.35	0.89	<0.01	<0.01	<0.01	0.07	0.03	0.08	5
IGB IF82D	Quartz-magnetite IF / Magnesian IF	53.23	0.07	42.28	2.60	2.60	2.03	0.02	<0.01	<0.01	0.02	-0.05	0.07	10
IGB IF82E	Quartz-magnetite IF / Magnesian IF	61.75	0.05	32.65	2.72	2.72	2.30	0.02	<0.01	<0.01	0.04	0.07	0.07	15
IGB IF82F	Quartz-magnetite IF / Magnesian IF	61.09	0.08	33.68	2.64	2.64	1.95	0.02	<0.01	<0.01	0.06	-0.04	0.06	10
IGB IF82G	Quartz-magnetite IF / Magnesian IF	52.11	0.23	41.86	0.32	3.55	1.86	0.01	<0.01	<0.01	0.05	-0.09	0.07	8
N 65° 10.325' W 49° 49.081'														
IGB IF31A	Quartz-magnetite IF / Magnesian IF	79.61	0.15	13.57	5.58	5.58	0.11	<0.01	<0.01	<0.01	<0.01	0.01	0.07	4
IGB IF31B	Quartz-magnetite IF / Magnesian IF	20.47	0.55	77.78	2.08	2.08	0.12	<0.01	<0.01	0.01	0.06	0.17	0.05	20
IGB IF31C	Quartz-magnetite IF / Magnesian IF	79.41	0.15	13.96	5.93	5.93	0.09	<0.01	<0.01	<0.01	<0.01	0.17	0.06	4
IGB IF31D	Quartz-magnetite IF / Magnesian IF	23.12	0.98	76.41	0.69	0.69	0.22	0.09	<0.01	0.02	0.12	0.04	0.06	20
IGB IF31E	Quartz-magnetite IF / Magnesian IF	55.34	0.59	40.37	4.12	4.12	0.12	<0.01	<0.01	0.01	0.03	0.15	0.06	20
IGB IF31F	Quartz-magnetite IF / Magnesian IF	17.16	0.93	82.06	1.38	1.38	0.11	0.01	0.02	0.03	0.05	0.13	0.06	20
IGB IF31G	Quartz-magnetite IF / Magnesian IF	57.88	0.24	37.30	4.52	4.52	0.09	<0.01	<0.01	<0.01	0.01	0.04	0.08	6
IGB IF31H	Quartz-magnetite IF / Magnesian IF	15.18	1.02	84.42	0.90	0.90	0.11	<0.01	0.02	0.03	0.03	0.16	0.07	20
IGB IF31K	Quartz-magnetite IF / Magnesian IF	83.04	0.30	12.90	4.40	4.40	0.08	<0.01	<0.01	<0.01	<0.01	0.15	0.08	4
IGB IF31L	Quartz-magnetite IF / Magnesian IF	44.40	0.50	55.15	0.96	0.96	0.10	<0.01	<0.01	0.01	0.03	0.05	0.07	20
IGB IF31M	Quartz-magnetite IF / Magnesian IF	86.42	0.09	9.29	3.97	3.97	0.06	<0.01	<0.01	<0.01	<0.01	0.19	0.08	4
IGB IF73A	Quartz-magnetite IF	54.27	0.08	37.70	1.72	1.72	2.58	0.02	<0.01	<0.01	0.07	-0.10	0.04	8
IGB IF73B1	Quartz-magnetite IF	62.68	0.04	32.20	1.20	1.20	1.79	<0.01	<0.01	<0.01	0.03	-0.03	0.06	8
IGB IF73B2	Quartz-magnetite IF	29.71	0.06	68.75	0.75	0.75	0.86	<0.01	<0.01	<0.01	0.04	0.03	0.06	8
IGB IF73C	Quartz-magnetite IF	39.65	0.04	57.50	0.93	0.93	1.24	<0.01	<0.01	<0.01	<0.01	0.06	0.04	5
IGB IF73D	Quartz-magnetite IF	38.56	0.07	58.83	0.85	0.85	1.12	<0.01	0.02	<0.01	0.04	-0.02	0.03	6
IGB IF73E	Quartz-magnetite IF	30.16	0.06	62.95	0.64	0.64	0.77	<0.01	0.02	<0.01	0.06	-0.08	0.07	12
IGB IF73F	Quartz-magnetite IF	77.09	0.05	10.06	2.49	2.49	3.96	0.01	0.01	<0.01	0.04	-0.04	0.06	20
IGB IF73G	Quartz-magnetite IF	31.04	0.30	67.75	0.05	0.61	0.22	<0.01	0.01	<0.01	0.03	0.16	0.06	5
IGB IF73H	Quartz-magnetite IF	85.25	0.06	11.29	0.67	0.67	1.02	0.01	<0.01	<0.01	0.01	0.10	0.06	8
IGB IF73I	Quartz-magnetite IF	26.43	0.12	64.34	0.23	0.23	0.12	0.04	<0.01	<0.01	0.04	-0.04	0.06	8
N 65° 12.495' W 49° 45.487'														
IGB IF77A	Quartz-magnetite IF	34.57	0.02	65.51	0.52	0.52	0.43	<0.01	<0.01	<0.01	0.20	0.09	0.06	15
IGB IF77B	Quartz-magnetite IF	74.54	0.07	17.74	1.59	1.59	2.49	0.02	<0.01	<0.01	<0.01	-0.12	0.07	10
IGB IF77C	Quartz-magnetite IF	59.21	0.02	36.24	1.23	1.23	1.86	<0.01	<0.01	<0.01	<0.01	-0.09	0.07	10
IGB IF77D	Quartz-magnetite IF	36.32	0.05	60.56	1.10	1.10	1.50	<0.01	<0.01	<0.01	<0.01	0.07	0.06	10
IGB IF77E	Quartz-magnetite IF	41.54	0.06	57.01	0.61	0.61	0.75	0.01	<0.01	<0.01	<0.01	0.07	0.06	7
IGB IF77F	Quartz-magnetite IF	34.69	0.06	62.83	0.92	0.92	1.23	<0.01	0.01	<0.01	<0.01	-0.01	0.06	6
IGB IF77G	Quartz-magnetite IF	76.73	0.05	9.89	2.64	2.64	4.26	0.02	<0.01	<0.01	0.01	-0.04	0.08	15
IGB IF77H	Quartz-magnetite IF	32.72	0.06	68.18	0.40	0.40	0.32	0.01	<0.01	<0.01	<0.01	0.08	0.06	15
IGB IF77K	Quartz-magnetite IF	91.10	0.04	5.60	0.58	0.58	0.93	0.02	<0.01	<0.01	<0.01	-0.07	0.08	4
97-Isua-1 668.74M A	Quartz-magnetite IF	40.39	0.22	59.64	0.08	0.08	0.06	<0.01	<0.01	<0.01	<0.01	-0.12	0.06	15
97-Isua-1 668.74M B	Quartz-magnetite IF	34.15	0.27	66.97	0.10	0.10	0.08	<0.01	<0.01	<0.01	0.01	-0.12	0.06	15
97-Isua-1 668.74M C	Quartz-magnetite IF	55.76	0.15	45.09	0.07	0.07	0.10	<0.01	<0.01	<0.01	0.03	-0.32	0.06	10
97-Isua-1 668.74M D	Quartz-magnetite IF	26.74	0.27	75.45	0.10	0.10	0.43	<0.01	<0.01	<0.01	0.26	-0.14	0.06	15
97-Isua-1 668.74M E	Quartz-magnetite IF	68.30	0.09	32.43	0.03	0.03	0.03	<0.01	<0.01	<0.01	0.01	-0.54	0.06	15
97-Isua-1 668.74M F	Quartz-magnetite IF	44.89	0.27	55.39	0.06	0.06	0.09	<0.01	<0.01	<0.01	0.05	0.08	0.07	20
97-Isua-1 668.74M G	Quartz-magnetite IF	35.73	0.40	63.44	0.13	0.13	0.09	<0.01	<0.01	<0.01	0.06	-0.16	0.06	15
97-Isua-1 668.74M H	Quartz-magnetite IF	27.65	0.35	71.38	0.03	0.03	0.02	<0.01	<0.01	0.01	<0.01	0.04	0.07	2
97-Isua-1 668.74M K	Quartz-magnetite IF	52.26	0.20	46.75	0.03	0.03	0.02	<0.01	<0.01	<0.01	<0.01	-0.43	0.06	15
IF-G 1	Quartz-magnetite IF	41.20	0.15	55.85	0.04	1.89	1.55	0.03	0.01	0.01	0.06	0.02	0.03	12
IF-G 2	Quartz-magnetite IF											0.02	0.06	15
IF-G 3	Quartz-magnetite IF											0.03	0.07	16
IF-G 4	Quartz-magnetite IF											0.05	0.06	20
IF-G 5	Quartz-magnetite IF											0.02	0.07	20
462912	Garnet-mica schist											-0.09	0.08	14
462919	Garnet-mica schist											-0.14	0.08	14
462915	Quartzo-feldspathic (felsic) schist											-0.12	0.07	12
462918	Quartzo-feldspathic (felsic) schist											-0.14	0.06	18
462901	metabasalt											-0.16	0.06	20
462949	metabasalt											-0.22	0.07	20
462947	metabasalt											-0.14	0.07	18

* n denotes the number of repeated mass spectrometrical runs
Major element concentrations in %, trace element concentrations (except where specified) in ppm
U*=authigenic U (measured U, corrected for 3.7 Ga decay and for detrital contamination using measured Th and a U/Th crustal value of 0.25)

Supplementary Table 1 | Major and selected trace elements, important element ratios and $\delta^{53}Cr$ values of BIF, metabasalt and metasediment samples from the IGB, West Greenland (continued)

Sample	Sc	Ti	V	Cr	Mn	Co	Ni	Cu	Zn	Ga	Rb	Sr	Y	Zr	Nb	Cs	Ba
	ppm	wt%	ppm	ppm	%	ppm	ppm	ppm	ppm	ppm	ppm	ppm	ppm	ppm	ppm	ppm	ppm
N 65° 10.354' W 49° 49.277'																	
IGB IF33A	0.25	0.000	20.38	0.41	0.025	2.05	16.97	0.91	13.12	0.11	0.037	0.222	1.364	0.160	0.102	0.003	0.074
IGB IF33B	2.40	0.006	19.22	2.81	0.014	2.03	20.74	24.24	10.75	0.91	0.178	0.541	1.201	0.211	0.160	0.012	1.391
IGB IF33C	0.75	0.001	22.65	1.78	0.017	1.64	15.96	1.61	10.37	0.48	0.086	0.316	0.910	0.104	0.098	0.006	0.433
IGB IF33D	0.88	0.003	25.71	5.09	0.010	1.78	18.71	5.07	6.80	0.94	0.163	0.397	1.339	0.235	0.143	0.012	0.874
IGB IF33E	0.58	0.000	26.16	1.21	0.036	2.36	20.94	0.49	19.87	0.23	0.037	0.203	0.610	0.154	0.118	0.003	0.366
IGB IF33F	0.11	0.004	23.90	1.56	0.016	2.37	25.12	1.43	11.44	1.15	0.158	0.571	2.348	0.430	0.216	0.014	1.133
IGB IF33G	0.90	0.001	26.10	1.48	0.032	2.44	23.35	1.26	19.40	0.48	0.104	0.323	1.117	0.225	0.145	0.008	0.996
N 65° 10.325' W 49° 49.081'																	
IGB IF32 A	0.31	0.007	2.54	5.90	0.028	2.04	26.86	2.58	13.53	1.21	0.402	1.582	2.037	3.804	0.309	0.061	11.437
IGB IF32 B	0.54	0.002	0.88	4.04	0.081	2.23	25.78	3.54	28.63	0.65	0.151	0.554	2.443	1.583	0.156	0.022	1.494
IGB IF32 C	0.37	0.005	2.17	3.84	0.044	2.09	23.92	1.98	18.20	1.18	0.219	0.602	1.643	2.408	0.195	0.034	1.595
IGB IF32 D	0.53	0.010	2.74	3.77	0.038	2.05	23.66	4.22	19.87	0.96	0.891	1.076	3.523	9.193	0.342	0.138	4.359
IGB IF82A	0.72	0.003	17.35	1.55	0.100	2.45	23.37	4.86	56.03	0.25	0.009	1.712	5.728	0.488	0.102	0.001	2.489
IGB IF82B	0.62	0.001	18.67	1.23	0.060	2.43	25.42	1.79	38.66	0.51	0.052	2.880	6.073	0.107	0.093	0.002	0.228
IGB IF82C	0.91	0.000	19.68	1.54	0.045	2.25	24.15	1.80	34.42	0.47	0.053	0.682	4.595	0.084	0.072	0.002	0.554
IGB IF82D	1.33	0.001	20.62	1.40	0.072	2.63	21.58	1.72	50.70	0.46	0.053	2.331	8.775	0.120	0.080	0.002	1.513
IGB IF82E	0.56	0.000	21.31	1.03	0.073	2.51	20.48	1.13	45.38	0.36	0.029	3.168	9.606	0.097	0.065	0.002	1.918
IGB IF82F	0.94	0.000	22.96	1.28	0.076	2.60	17.10	1.81	47.42	0.36	0.043	2.326	7.240	0.430	0.070	0.002	3.533
IGB IF82G	0.69	0.001	19.89	1.25	0.043	1.90	16.18	8.00	37.66	0.39	0.043	1.195	2.831	0.108	0.053	0.001	1.725
N 65° 10.325' W 49° 49.081'																	
IGB IF31A	1.59	0.001	0.24	0.34	0.103	2.14	25.24	7.65	31.91	0.32	0.059	0.702	0.946	0.198	0.133	0.005	1.120
IGB IF31B	0.64	0.006	2.24	12.15	0.040	2.84	36.07	2.79	19.31	1.95	0.375	1.194	5.470	6.785	0.384	0.054	3.098
IGB IF31C	1.65	0.001	0.55	0.12	0.112	2.27	28.01	7.11	31.85	0.26	0.032	0.465	0.742	0.188	0.104	0.018	0.943
IGB IF31D	0.67	0.016	3.16	8.27	0.014	3.01	37.95	3.40	12.07	2.44	0.509	2.497	5.117	6.263	0.584	0.062	4.896
IGB IF31E	1.29	0.006	1.29	1.47	0.071	2.68	35.61	2.14	27.04	1.20	0.256	0.993	2.828	1.070	0.276	0.036	2.467
IGB IF31F	0.27	0.011	3.70	13.17	0.022	2.98	41.31	3.62	15.30	2.20	0.697	1.734	5.274	13.082	0.538	0.095	4.413
IGB IF31G	1.80	0.002	0.22	0.54	0.082	1.89	23.71	1.89	30.71	0.41	0.074	0.776	1.788	0.363	0.148	0.010	1.913
IGB IF31H	0.45	0.015	5.37	13.12	0.013	3.13	45.78	3.42	12.27	2.31	0.627	1.653	6.635	11.989	0.608	0.088	4.016
IGB IF31K	1.03	0.001	0.50	0.17	0.077	1.76	22.41	2.79	24.61	0.27	0.066	0.627	1.725	0.163	0.146	0.008	0.965
IGB IF31L	0.45	0.008	3.37	5.58	0.019	2.32	31.91	7.00	10.12	1.48	0.368	1.345	4.303	5.168	0.298	0.057	2.234
IGB IF31M	0.76	0.000	0.84	0.11	0.091	1.64	20.77	2.07	18.54	0.13	0.036	0.400	2.802	0.136	0.070	0.005	1.015
IGB IF73A	0.41	0.001	18.95	1.73	0.027	1.83	13.13	0.44	33.15	0.40	0.129	14.676	8.113	0.143	0.053	0.015	5.847
IGB IF73B1	1.23	0.000	22.85	1.12	0.020	1.71	6.84	0.99	14.24	0.41	0.108	10.532	4.785	0.090	0.046	0.015	5.480
IGB IF73B2	0.35	0.002	21.82	1.48	0.020	3.21	13.51	1.44	31.12	0.92	0.304	5.772	4.669	0.161	0.122	0.048	8.630
IGB IF73C	0.99	0.000	24.78	1.58	0.021	2.60	11.69	2.02	26.55	0.75	0.148	7.051	4.456	0.068	0.076	0.029	6.606
IGB IF73D	0.21	0.001	20.32	2.30	0.021	2.81	16.52	1.22	25.79	0.79	0.191	6.596	6.462	0.067	0.058	0.028	6.646
IGB IF73E	0.30	0.002	24.73	3.70	0.020	3.67	22.93	2.02	33.97	1.03	0.206	4.996	9.491	0.141	0.099	0.035	5.682
IGB IF73F	0.27	0.000	27.33	1.39	0.038	0.43	2.64	0.71	5.17	0.18	0.052	22.079	5.290	0.069	0.045	0.004	5.387
IGB IF73G	0.57	0.002	24.77	3.99	0.016	4.10	25.83	1.38	38.22	1.10	0.305	1.584	5.783	0.118	0.113	0.039	7.455
IGB IF73H	1.61	0.000	29.75	1.80	0.012	0.66	3.36	2.06	19.93	0.22	0.096	6.569	6.120	0.063	0.028	0.007	18.420
IGB IF73I	0.77	0.002	23.69	2.96	0.011	3.17	22.88	2.17	26.33	0.97	0.277	1.232	26.044	0.415	0.161	0.043	12.976
N 65° 12.495' W 49° 45.487'																	
IGB IF77A	0.53	0.002	0.47	2.43	0.010	2.78	23.17	0.14	19.38	0.71	0.116	2.920	8.893	0.509	0.074	0.025	3.589
IGB IF77B	4.26	0.000	0.52	0.45	0.027	1.28	7.28	0.40	7.97	0.20	0.054	9.773	3.274	0.078	0.016	0.003	5.081
IGB IF77C	2.15	0.000	0.43	0.48	0.027	2.50	10.14	0.25	15.56	0.51	0.135	9.848	3.401	0.143	0.023	0.018	4.692
IGB IF77D	0.66	0.001	0.23	0.60	0.027	3.28	12.89	0.25	23.88	0.80	0.144	7.664	5.619	0.175	0.038	0.029	5.848
IGB IF77E	0.88	0.002	0.22	0.55	0.018	2.95	13.34	0.07	22.09	0.70	0.151	4.121	2.603	0.170	0.035	0.028	5.831
IGB IF77F	0.60	0.002	0.11	2.21	0.023	3.26	20.12	0.34	23.67	0.76	0.200	6.282	6.283	0.212	0.031	0.025	5.101
IGB IF77G	5.01	0.000	0.68	0.12	0.038	0.75	2.31	0.50	3.88	0.12	0.068	21.551	4.794	0.075	0.029	0.002	2.824
IGB IF77H	0.37	0.002	0.03	2.84	0.018	4.13	26.35	0.18	29.51	0.99	0.231	2.202	8.710	0.328	0.046	0.035	5.613
IGB IF77K	2.49	0.000	0.67	0.07	0.010	0.64	1.43	0.30	11.05	0.10	0.061	5.802	3.730	0.090	0.013	0.004	4.739
97-Isua-1 668.74M A	1.41	0.001	2.35	5.38	0.004	7.96	27.04	43.55	6.24	1.27	0.240	1.270	6.735	1.605	0.193	0.081	50.991
97-Isua-1 668.74M B	0.65	0.004	2.53	5.34	0.004	4.07	24.03	28.03	3.92	1.29	0.248	1.259	4.709	1.455	0.180	0.079	16.171
97-Isua-1 668.74M C	1.85	0.002	1.30	3.33	0.004	4.32	17.96	33.00	3.70	0.87	0.207	1.239	4.697	0.978	0.099	0.057	16.612
97-Isua-1 668.74M D	1.36	0.001	1.78	5.36	0.004	3.98	24.96	20.76	4.08	1.57	0.336	2.875	6.710	1.521	0.175	0.118	14.153
97-Isua-1 668.74M E	1.16	0.001	0.86	3.48	0.003	7.42	15.03	55.32	2.73	0.45	0.109	0.869	3.178	0.483	0.063	0.024	17.729
97-Isua-1 668.74M F	0.64	0.004	1.56	5.12	0.004	4.52	25.73	48.30	3.89	1.24	0.245	1.342	4.237	1.368	0.132	0.131	18.707
97-Isua-1 668.74M G	0.32	0.005	1.66	4.10	0.005	4.55	31.09	78.53	5.17	1.43	0.370	1.578	3.196	3.179	0.156	0.189	22.688
97-Isua-1 668.74M H	0.89	0.006	2.72	5.55	0.005	4.83	24.78	47.73	3.33	1.54	0.217	1.319	2.636	1.948	0.214	0.091	15.413
97-Isua-1 668.74M K	1.80	0.003	1.17	3.63	0.003	4.33	18.45	35.91	3.46	1.08	0.219	0.932	3.471	1.154	0.142	0.062	7.825
IF-G 1	0.40	0.002	18.32	3.24	0.021	18.82	17.53	6.06	15.61	0.57	0.311	3.487	9.618	0.455	0.158	0.053	2.621
IF-G 2	1.17	0.002	21.28	3.24	0.022	19.08	17.68	5.98	14.20	0.59	0.330	3.636	9.467	0.562	0.169	0.054	3.113
IF-G 3	0.76	0.002	21.39	3.56	0.022	19.34	17.98	6.84	14.10	0.58	0.312	3.544	9.523	0.428	0.169	0.052	2.851
IF-G 4	0.78	0.001	21.24	3.30	0.021	18.74	17.32	5.78	13.97	0.55	0.305	3.416	9.316	0.409	0.158	0.051	2.460
IF-G 5	0.65	0.002	24.62	3.40	0.023	20.66	18.85	8.00	16.16	0.62	0.353	4.011	11.122	0.510	0.198	0.059	2.715
462912				166													
462919				77													
462915				9.58													
462918				29.05													
462901				959													
462949				199													
462947				189													

* n denotes the number of repeat
Major element concentrations in
U*=authigenic U (measured U, cc

Supplementary Table 1 | ISupplementary Table 1 | Major and selected trace elements, important element ratios and $\delta^{53}Cr$ values of BIF, metabasalt and metasedimmetabasalt and metasediment samples from the IGB, West Greenland (continued)

Sample	La	Ce	Pr	Nd	Sm	Eu	Gd	Tb	Dy	Y	Ho	Er	Tm	Yb	Lu	Hf	Ta	Pb
	ppm	ppm	ppm	ppm	ppm	ppm	ppm	ppm	ppm	ppm	ppm	ppm	ppm	ppm	ppm	ppm	ppm	ppm
N 65° 10.354' W 49° 49.277'																		
IGB IF33A	0.232	0.578	0.047	0.206	0.054	0.051	0.112	0.020	0.157	1.364	0.040	0.118	0.019	0.121	0.020	0.009	0.004	0.120
IGB IF33B	1.149	1.799	0.217	0.917	0.179	0.131	0.190	0.025	0.134	1.201	0.028	0.083	0.012	0.072	0.012	0.007	0.000	2.065
IGB IF33C	0.844	1.355	0.170	0.732	0.158	0.116	0.171	0.021	0.104	0.910	0.022	0.066	0.009	0.063	0.010	0.008	0.001	0.095
IGB IF33D	1.304	2.047	0.250	1.065	0.214	0.162	0.241	0.028	0.174	1.339	0.034	0.089	0.013	0.073	0.011	0.008	0.000	0.390
IGB IF33E	0.308	0.489	0.060	0.248	0.054	0.044	0.067	0.009	0.063	0.610	0.013	0.049	0.007	0.056	0.010	0.008	0.009	0.091
IGB IF33F	0.609	1.083	0.143	0.661	0.187	0.168	0.271	0.042	0.273	2.348	0.060	0.165	0.027	0.149	0.023	0.016	0.015	0.098
IGB IF33G	0.384	0.651	0.077	0.317	0.081	0.072	0.130	0.020	0.120	1.117	0.027	0.080	0.012	0.087	0.015	0.010	0.003	0.100
N 65° 10.325' W 49° 49.081'																		
IGB IF32 A	3.014	5.520	0.594	2.453	0.495	0.333	0.531	0.064	0.327	2.037	0.058	0.170	0.023	0.148	0.023	0.099	0.021	0.746
IGB IF32 B	2.389	4.341	0.459	1.847	0.362	0.248	0.402	0.055	0.324	2.443	0.065	0.214	0.031	0.218	0.034	0.040	0.014	0.719
IGB IF32 C	2.930	5.089	0.532	2.117	0.401	0.260	0.435	0.049	0.252	1.643	0.046	0.140	0.019	0.129	0.019	0.056	0.017	0.668
IGB IF32 D	5.243	9.071	0.932	3.668	0.681	0.438	0.757	0.088	0.470	3.523	0.092	0.285	0.038	0.255	0.039	0.259	0.020	0.795
IGB IF82A	1.841	3.073	0.323	1.322	0.259	0.203	0.431	0.067	0.478	5.728	0.115	0.374	0.058	0.375	0.072	0.017	0.024	1.383
IGB IF82B	0.904	1.467	0.185	0.868	0.225	0.191	0.382	0.068	0.481	6.073	0.117	0.371	0.056	0.329	0.057	0.008	0.008	0.724
IGB IF82C	0.810	1.264	0.163	0.746	0.183	0.173	0.323	0.052	0.400	4.595	0.094	0.296	0.042	0.272	0.040	0.007	0.014	0.575
IGB IF82D	1.535	2.513	0.322	1.473	0.345	0.337	0.636	0.100	0.752	8.775	0.179	0.569	0.083	0.512	0.085	0.010	0.009	0.535
IGB IF82E	1.567	2.521	0.336	1.553	0.387	0.357	0.651	0.106	0.761	9.606	0.189	0.592	0.085	0.534	0.092	0.008	0.022	0.471
IGB IF82F	1.239	1.904	0.233	1.027	0.252	0.228	0.429	0.071	0.545	7.240	0.140	0.440	0.064	0.405	0.071	0.016	0.003	0.974
IGB IF82G	0.555	0.866	0.100	0.435	0.101	0.093	0.175	0.030	0.224	2.831	0.054	0.179	0.027	0.160	0.027	0.005	0.002	5.207
N 65° 10.325' W 49° 49.081'																		
IGB IF31A	0.251	0.578	0.052	0.230	0.056	0.044	0.085	0.016	0.117	0.946	0.028	0.085	0.016	0.116	0.018	0.008	0.011	1.157
IGB IF31B	2.230	4.198	0.466	1.983	0.496	0.341	0.616	0.095	0.576	5.470	0.128	0.391	0.057	0.380	0.056	0.129	0.136	0.776
IGB IF31C	0.922	2.071	0.189	0.726	0.155	0.084	0.129	0.016	0.099	0.742	0.021	0.071	0.013	0.099	0.016	0.009	0.125	1.720
IGB IF31D	2.209	4.620	0.533	2.364	0.655	0.456	0.790	0.114	0.649	5.117	0.124	0.337	0.048	0.274	0.042	0.136	0.030	0.785
IGB IF31E	0.771	1.450	0.187	0.881	0.259	0.187	0.343	0.052	0.335	2.828	0.072	0.217	0.035	0.228	0.035	0.032	0.107	0.659
IGB IF31F	2.171	4.239	0.470	2.017	0.491	0.344	0.630	0.093	0.595	5.274	0.130	0.385	0.058	0.359	0.056	0.304	0.042	1.235
IGB IF31G	0.452	0.825	0.103	0.469	0.123	0.091	0.181	0.029	0.202	1.788	0.047	0.146	0.025	0.180	0.028	0.013	0.019	1.421
IGB IF31H	2.480	4.658	0.530	2.268	0.545	0.372	0.717	0.113	0.740	6.635	0.164	0.480	0.070	0.429	0.073	0.280	0.103	0.536
IGB IF31K	0.216	0.540	0.051	0.211	0.067	0.057	0.133	0.025	0.198	1.725	0.049	0.152	0.028	0.174	0.029	0.007	0.014	1.457
IGB IF31L	1.395	2.755	0.305	1.352	0.364	0.251	0.486	0.077	0.511	4.303	0.112	0.319	0.049	0.290	0.046	0.107	0.016	1.196
IGB IF31M	0.053	0.133	0.015	0.083	0.044	0.050	0.114	0.026	0.233	2.802	0.065	0.224	0.037	0.230	0.038	0.009	0.021	1.389
IGB IF73A	5.737	6.866	0.712	2.651	0.401	0.275	0.657	0.089	0.609	8.113	0.152	0.442	0.061	0.366	0.064	0.009	0.008	5.213
IGB IF73B1	0.975	1.373	0.168	0.724	0.187	0.124	0.325	0.050	0.356	4.785	0.089	0.274	0.040	0.250	0.041	0.005	0.008	4.546
IGB IF73B2	1.097	1.553	0.168	0.699	0.160	0.109	0.278	0.047	0.337	4.669	0.087	0.281	0.045	0.278	0.049	0.006	0.011	4.366
IGB IF73C	0.839	1.227	0.139	0.676	0.144	0.105	0.263	0.043	0.327	4.456	0.085	0.267	0.040	0.255	0.045	0.004	0.002	3.990
IGB IF73D	1.331	1.758	0.195	0.876	0.199	0.143	0.359	0.062	0.487	6.462	0.127	0.389	0.057	0.359	0.061	0.006	0.000	3.421
IGB IF73E	2.829	3.487	0.352	1.466	0.306	0.214	0.549	0.095	0.716	9.491	0.180	0.561	0.085	0.516	0.086	0.008	0.002	3.042
IGB IF73F	0.659	1.073	0.148	0.717	0.186	0.151	0.326	0.053	0.367	5.290	0.098	0.328	0.050	0.306	0.059	0.003	0.001	5.617
IGB IF73G	1.383	1.780	0.185	0.745	0.175	0.125	0.320	0.054	0.446	5.783	0.113	0.357	0.058	0.350	0.056	0.006	0.005	2.064
IGB IF73H	0.699	1.063	0.155	0.713	0.189	0.149	0.355	0.060	0.491	6.120	0.129	0.409	0.059	0.355	0.060	0.004	0.002	2.195
IGB IF73I	3.492	5.319	0.626	2.909	0.798	0.624	1.519	0.265	2.036	26.044	0.518	1.647	0.241	1.415	0.233	0.023	0.171	2.905
N 65° 12.495' W 49° 45.487'																		
IGB IF77A	1.336	2.325	0.294	1.415	0.339	0.254	0.621	0.088	0.602	8.893	0.154	0.430	0.059	0.342	0.056	0.018	0.011	1.202
IGB IF77B	0.602	1.047	0.118	0.550	0.132	0.110	0.242	0.037	0.252	3.274	0.067	0.187	0.025	0.150	0.025	0.003	0.003	3.917
IGB IF77C	0.551	0.885	0.102	0.491	0.116	0.091	0.219	0.034	0.236	3.401	0.062	0.191	0.028	0.181	0.031	0.004	0.002	3.838
IGB IF77D	0.666	1.119	0.143	0.694	0.184	0.131	0.331	0.053	0.406	5.619	0.105	0.324	0.047	0.302	0.049	0.006	0.028	3.988
IGB IF77E	0.469	0.729	0.082	0.352	0.084	0.057	0.134	0.024	0.173	2.603	0.048	0.149	0.022	0.154	0.027	0.006	0.005	2.847
IGB IF77F	0.956	1.438	0.166	0.741	0.191	0.143	0.359	0.062	0.465	6.283	0.123	0.379	0.054	0.354	0.057	0.009	0.015	2.348
IGB IF77G	0.561	0.985	0.127	0.611	0.181	0.135	0.295	0.050	0.343	4.794	0.092	0.284	0.044	0.297	0.056	0.004	0.061	5.055
IGB IF77H	0.890	1.405	0.173	0.813	0.235	0.179	0.456	0.085	0.624	8.710	0.171	0.527	0.080	0.491	0.082	0.012	0.010	1.822
IGB IF77K	0.808	1.181	0.134	0.564	0.129	0.102	0.228	0.039	0.270	3.730	0.074	0.234	0.036	0.225	0.038	0.004	0.006	1.761
97-Isua-1 668.74M A	5.296	9.038	1.037	4.301	0.766	0.384	0.858	0.117	0.695	6.735	0.144	0.433	0.056	0.346	0.056	0.044	0.010	1.076
97-Isua-1 668.74M B	4.018	7.136	0.767	3.140	0.594	0.289	0.643	0.084	0.491	4.709	0.102	0.289	0.042	0.248	0.035	0.037	0.033	0.873
97-Isua-1 668.74M C	2.258	4.061	0.453	1.934	0.407	0.238	0.526	0.074	0.467	4.697	0.102	0.280	0.042	0.242	0.039	0.027	0.008	1.192
97-Isua-1 668.74M D	3.162	5.487	0.688	3.245	0.810	0.451	1.025	0.134	0.766	6.710	0.151	0.410	0.056	0.317	0.047	0.041	0.037	0.864
97-Isua-1 668.74M E	0.658	1.267	0.158	0.724	0.204	0.135	0.323	0.051	0.311	3.178	0.072	0.204	0.029	0.171	0.025	0.015	0.029	1.517
97-Isua-1 668.74M F	1.211	2.483	0.289	1.343	0.360	0.208	0.466	0.066	0.423	4.237	0.092	0.258	0.037	0.231	0.033	0.040	0.186	0.910
97-Isua-1 668.74M G	1.773	3.450	0.442	2.000	0.422	0.218	0.468	0.063	0.376	3.196	0.076	0.211	0.030	0.184	0.028	0.086	0.010	1.027
97-Isua-1 668.74M H	1.120	2.376	0.318	1.534	0.395	0.211	0.423	0.056	0.301	2.636	0.061	0.174	0.024	0.156	0.023	0.054	0.017	1.831
97-Isua-1 668.74M K	1.992	3.978	0.418	1.830	0.386	0.206	0.437	0.061	0.369	3.471	0.082	0.221	0.033	0.198	0.032	0.036	0.010	0.977
IF-G 1	2.843	4.081	0.442	1.901	0.417	0.363	0.682	0.109	0.813	9.618	0.194	0.617	0.089	0.547	0.092	0.016	0.183	2.866
IF-G 2	2.409	3.636	0.397	1.714	0.384	0.340	0.632	0.106	0.796	9.467	0.191	0.608	0.091	0.546	0.088	0.020	0.195	2.780
IF-G 3	2.350	3.467	0.397	1.725	0.389	0.355	0.656	0.106	0.803	9.523	0.196	0.620	0.092	0.548	0.091	0.019	0.180	2.926
IF-G 4	2.410	3.568	0.409	1.720	0.372	0.335	0.617	0.105	0.845	9.316	0.187	0.597	0.088	0.531	0.087	0.023	0.172	2.615
IF-G 5	2.875	4.182	0.476	2.039	0.463	0.411	0.759	0.126	0.943	11.122	0.224	0.701	0.103	0.626	0.097	0.019	0.205	3.335
462912																		
462919																		
462915																		
462918																		
462901																		
462949																		
462947																		

* n denotes the number of repeat
Major element concentrations in
U*=authigenic U (measured U, cc

Supplementary Table 1 | I
Supplementary Table 1 | Major and selected trace elements, important element ratios and $\delta^{53}Cr$ values of BIF, metabasalt and metasediment samples from the IGB, West Greenland (continued)
metabasalt and metasedi

Sample	Th	U	Mo	Fe_2O_3/SiO_2	ΣREE+Y	$(Sm/Yb)_{PAAS}$	$(Nd/Yb)_{PAAS}$	Eu/Eu*	Gd/Gd*	Ce/Ce*	Pr/Pr*	La/La*	Y/Ho	U/Th	U*
	ppm	ppm	ppm												ppm
N 65° 10.354' W 49° 49.277'															
IGB IF33A	0.004	0.003	0.022	0.208	1.775	0.225	0.141	3.18	1.18	1.27	0.80	1.58	34.29	0.91	0.004
IGB IF33B	0.025	0.027	0.191	0.883	4.949	1.271	1.065	3.77	1.27	0.83	0.99	1.53	42.65	1.10	0.039
IGB IF33C	0.020	0.015	0.108	0.480	3.840	1.270	0.963	3.86	1.35	0.82	1.00	1.50	41.82	0.77	0.019
IGB IF33D	0.030	0.028	0.160	0.923	5.704	1.490	1.216	3.97	1.40	0.82	0.99	1.55	39.33	0.92	0.038
IGB IF33E	0.010	0.008	0.095	0.271	1.478	0.485	0.368	3.91	1.28	0.83	1.01	1.38	47.08	0.82	0.010
IGB IF33F	0.013	0.010	0.216	1.872	3.861	0.637	0.369	3.84	1.23	0.85	0.98	1.69	38.81	0.76	0.012
IGB IF33G	0.006	0.006	0.191	1.039	2.073	0.478	0.305	3.62	1.26	0.87	0.99	1.38	41.43	0.96	0.008
N 65° 10.325' W 49° 49.081'															
IGB IF32 A	0.071	0.030	-	1.586	13.755	1.697	1.375	3.53	1.34	0.95	0.95	1.38	34.83	0.42	0.025
IGB IF32 B	0.096	0.021	-	0.492	10.989	0.845	0.706	3.42	1.25	0.95	0.95	1.33	37.32	0.22	0.000
IGB IF32 C	0.096	0.023	-	1.002	12.418	1.585	1.369	3.47	1.41	0.93	0.95	1.37	35.77	0.24	0.003
IGB IF32 D	0.115	0.041	-	0.721	22.058	1.355	1.195	3.39	1.39	0.94	0.95	1.37	38.35	0.36	0.028
IGB IF82A	0.013	0.013	0.184	1.208	8.990	0.351	0.293	3.15	1.26	0.91	0.94	1.52	49.71	0.98	0.018
IGB IF82B	0.016	0.011	0.450	0.782	5.702	0.346	0.219	3.16	1.14	0.83	0.95	2.01	52.00	0.67	0.013
IGB IF82C	0.018	0.009	0.196	0.626	4.857	0.342	0.228	3.64	1.25	0.80	0.97	1.89	49.13	0.51	0.010
IGB IF82D	0.021	0.013	0.131	0.794	9.439	0.342	0.239	3.71	1.27	0.82	0.97	1.79	48.94	0.62	0.015
IGB IF82E	0.018	0.014	0.115	0.529	9.730	0.368	0.242	3.60	1.22	0.80	0.98	1.81	50.95	0.81	0.018
IGB IF82F	0.018	0.012	0.421	0.551	7.047	0.315	0.211	3.48	1.21	0.81	0.97	1.75	51.86	0.68	0.015
IGB IF82G	0.010	0.007	0.181	0.803	3.026	0.320	0.226	3.42	1.16	0.84	0.95	1.76	52.19	0.66	0.008
N 65° 10.325' W 49° 49.081'															
IGB IF31A	0.011	0.011	0.037	0.170	1.693	0.246	0.165	3.00	1.06	1.16	0.84	1.57	33.81	1.02	0.016
IGB IF31B	0.076	0.061	1.734	3.800	12.013	0.663	0.434	3.15	1.19	0.95	0.95	1.41	42.70	0.80	0.078
IGB IF31C	0.011	0.010	0.026	0.176	4.612	0.797	0.610	3.02	1.19	1.14	0.90	1.13	34.84	0.89	0.013
IGB IF31D	0.107	0.049	0.442	3.305	13.216	1.214	0.717	3.31	1.23	0.98	0.94	1.39	41.29	0.45	0.044
IGB IF31E	0.041	0.017	0.099	0.729	5.051	0.576	0.321	3.25	1.23	0.88	0.96	1.75	39.41	0.41	0.014
IGB IF31F	0.143	0.164	0.784	4.782	12.038	0.695	0.467	3.22	1.23	0.97	0.94	1.39	40.42	1.15	0.237
IGB IF31G	0.034	0.037	0.031	0.644	2.901	0.346	0.216	3.08	1.19	0.88	0.96	1.64	37.93	1.10	0.053
IGB IF31H	0.136	0.160	1.216	5.561	13.638	0.645	0.439	3.02	1.18	0.94	0.96	1.40	40.50	1.18	0.232
IGB IF31K	0.012	0.005	0.016	0.155	1.931	0.196	0.101	2.81	1.11	1.19	0.88	1.18	35.10	0.46	0.005
IGB IF31L	0.048	0.054	0.205	1.242	8.312	0.637	0.387	3.03	1.18	0.97	0.93	1.53	38.47	1.11	0.077
IGB IF31M	0.008	0.007	0.021	0.107	1.347	0.098	0.030	2.80	0.97	1.08	0.83	6.21	42.83	0.88	0.009
IGB IF73A	0.011	0.008	0.111	0.695	19.082	0.556	0.602	2.94	1.40	0.75	0.98	1.76	53.32	0.72	0.010
IGB IF73B1	0.009	0.008	0.052	0.514	4.975	0.381	0.241	2.61	1.29	0.77	0.99	1.77	53.84	0.92	0.011
IGB IF73B2	0.019	0.021	0.064	2.314	5.189	0.292	0.209	2.56	1.18	0.82	0.95	1.80	53.73	1.07	0.029
IGB IF73C	0.011	0.012	0.030	1.450	4.454	0.287	0.220	2.74	1.24	0.82	0.89	2.93	52.52	1.09	0.017
IGB IF73D	0.020	0.011	0.037	1.526	6.402	0.281	0.203	2.61	1.17	0.78	0.92	2.41	51.01	0.53	0.011
IGB IF73E	0.039	0.011	0.029	2.087	11.442	0.301	0.237	2.56	1.17	0.77	0.91	2.25	52.76	0.28	0.004
IGB IF73F	0.010	0.003	0.060	0.130	4.519	0.309	0.195	3.13	1.24	0.79	0.97	2.14	54.07	0.28	0.001
IGB IF73G	0.026	0.012	0.029	2.182	6.148	0.254	0.177	2.62	1.20	0.78	0.95	1.90	51.31	0.47	0.012
IGB IF73H	0.015	0.004	0.082	0.132	4.885	0.270	0.167	2.84	1.20	0.74	1.02	1.71	47.62	0.24	0.001
IGB IF73I	0.036	0.021	0.069	2.434	21.643	0.287	0.171	2.76	1.18	0.82	0.93	2.23	50.25	0.58	0.023
N 65° 12.495' W 49° 45.487'															
IGB IF77A	0.012	0.010	0.059	1.895	8.314	0.503	0.344	3.00	1.38	0.86	0.94	2.14	57.77	0.90	0.014
IGB IF77B	0.004	0.003	0.036	0.238	3.544	0.447	0.306	3.19	1.29	0.90	0.91	2.02	49.22	0.83	0.004
IGB IF77C	0.005	0.003	0.026	0.612	3.221	0.326	0.225	2.95	1.30	0.85	0.91	2.49	54.59	0.50	0.003
IGB IF77D	0.011	0.005	0.036	1.667	4.552	0.309	0.191	2.70	1.25	0.84	0.94	2.27	53.62	0.45	0.004
IGB IF77E	0.011	0.005	0.032	1.372	2.506	0.278	0.190	2.57	1.11	0.85	0.95	1.76	54.64	0.49	0.005
IGB IF77F	0.015	0.007	0.031	1.811	5.486	0.274	0.174	2.66	1.18	0.83	0.94	1.99	51.13	0.45	0.006
IGB IF77G	0.010	0.005	0.021	0.129	4.060	0.309	0.171	2.90	1.18	0.85	0.95	2.06	52.37	0.47	0.005
IGB IF77H	0.026	0.009	0.044	2.084	6.210	0.243	0.138	2.56	1.12	0.82	0.94	2.14	51.01	0.35	0.006
IGB IF77K	0.009	0.010	0.093	0.061	4.060	0.293	0.209	2.92	1.18	0.82	0.96	1.74	50.70	1.15	0.015
97-Isua-1 668.74M A	0.066	0.075	0.203	1.477	23.527	1.126	1.035	2.50	1.25	0.89	0.98	1.41	46.88	1.14	0.107
97-Isua-1 668.74M B	0.086	0.100	0.247	1.961	17.879	1.215	1.051	2.49	1.28	0.93	0.95	1.39	46.24	1.16	0.145
97-Isua-1 668.74M C	0.048	0.053	0.179	0.809	11.123	0.854	0.665	2.73	1.28	0.92	0.95	1.49	46.15	1.11	0.076
97-Isua-1 668.74M D	0.055	0.063	0.246	2.822	16.749	1.300	0.853	2.70	1.34	0.86	0.95	1.95	44.34	1.15	0.090
97-Isua-1 668.74M E	0.033	0.033	0.206	0.475	4.331	0.606	0.352	2.70	1.24	0.91	0.96	1.56	44.21	1.00	0.045
97-Isua-1 668.74M F	0.060	0.053	0.316	1.234	7.499	0.791	0.483	2.69	1.27	0.97	0.93	1.66	46.12	0.89	0.071
97-Isua-1 668.74M G	0.036	0.036	0.384	1.776	9.741	1.164	0.903	2.59	1.26	0.90	0.98	1.45	41.98	1.01	0.050
97-Isua-1 668.74M H	0.039	0.040	0.278	2.582	7.171	1.283	0.817	2.73	1.27	0.91	0.96	1.66	43.54	1.02	0.056
97-Isua-1 668.74M K	0.056	0.042	0.160	0.895	10.241	0.989	0.768	2.63	1.24	1.00	0.91	1.53	42.38	0.76	0.054
IF-G 1	0.044	0.023	0.489	1.356	13.190	0.387	0.289	3.47	1.23	0.82	0.93	1.95	49.57	0.52	0.024
IF-G 2	0.043	0.023	0.530		11.938	0.358	0.261	3.43	1.18	0.85	0.93	1.87	49.53	0.53	0.023
IF-G 3	0.043	0.021	0.515		11.795	0.361	0.262	3.56	1.22	0.82	0.95	1.85	48.63	0.49	0.021
IF-G 4	0.045	0.025	0.468		11.870	0.355	0.269	3.47	1.17	0.82	0.97	1.68	49.86	0.57	0.028
IF-G 5	0.049	0.026	0.576		14.024	0.376	0.271	3.48	1.20	0.81	0.96	1.82	49.72	0.53	0.027
462912	0.460	0.150												0.33	
462919	5.430	0.850												0.16	
462915	6.910	1.150												0.17	
462918	8.050	1.570												0.20	
462901	0.110	0.031												0.28	
462949	0.180	0.041												0.23	
462947	0.240	0.031												0.13	

* n denotes the number of repeat
Major element concentrations in
U*=authigenic U (measured U, cc

Supplementary Table 2 | Summary of statistical comparisons (Welch's t-test for d53Cr; one sample t-test for U/Th ratios)

Sample	Average d53Cr	sd	n	against	t	p	different
IGB IF33	0.15	0.15	7	IMR	4.9	<0.0001	yes
IGB IF32	0.10	0.04	4	IMR	10.0	<0.0001	yes
IGB IF82	-0.02	0.05	7	IMR	4.9	0.0004	yes
IGB IF31	0.11	0.07	11	IMR	10.5	<0.0001	yes
IGB IF73	0.00	0.08	10	IMR	5.0	0.0003	yes
IGB IF77	0.00	0.08	9	IMR	4.6	0.0006	yes
IF-G	0.03	0.01	5	IMR	10.7	<0.0001	yes
97-Isua-1 668.74M	-0.19	0.21	9	IMR	-0.6	0.5505	no
All*	0.05	0.10	53	IMR	6.6	0.0003	yes
MISE	-0.12	0.05	24	IMR	1.3	0.2348	no
All*	0.02	0.15	53	MISE	7.0	<0.0001	yes

MISE = Mean Igneous Silicate Earth[15], IMR= Isua metabasalts and clastic metasediments (Supplementary Table 1).
* except sample 97-Isua-1 668.74M (weathered!)

Sample	Average U/Th	sd	n	against	t	p	different
IGB IF33	0.9	0.1	7	AC	14.0	<0.0001	yes
IGB IF32	0.3	0.1	4	AC	1.1	0.3517	no
IGB IF82	0.7	0.1	7	AC	8.0	0.0002	yes
IGB IF31	0.8	0.3	11	AC	6.9	<0.0001	yes
IGB IF73	0.6	0.3	10	AC	3.3	0.0092	yes
97-Isua-1 668.74M	0.6	0.3	9	AC	2.9	0.007	yes
IGB IF77	1.0	0.1	5	AC	13.0	0.0199	yes
IF-G	0.5	0.0	5	AC	19.2	<0.0001	yes
All	0.7	0.3	58	AC	11.7	<0.0001	yes

AC= Archean Crust U/Th (0.2564[20])
